# Nanobodies: a promising approach to treatment of viral diseases

**DOI:** 10.3389/fimmu.2023.1303353

**Published:** 2024-01-23

**Authors:** Vitória Meneghetti Minatel, Carlos Roberto Prudencio, Benedito Barraviera, Rui Seabra Ferreira

**Affiliations:** ^1^ Center for the Study of Venoms and Venomous Animals (CEVAP), São Paulo State University (UNESP—Univ Estadual Paulista), Botucatu, São Paulo, Brazil; ^2^ Immunology Center, Adolfo Lutz Institute, São Paulo, São Paulo, Brazil; ^3^ Graduate Program in Tropical Diseases, Botucatu Medical School (FMB), São Paulo State University (UNESP—Univ Estadual Paulista), Botucatu, São Paulo, Brazil

**Keywords:** camelids, heavy chain antibodies, single domain antibodies, immune library, phage display, VHH, neutralizing antibodies

## Abstract

Since their discovery in the 1990s, heavy chain antibodies have garnered significant interest in the scientific community. These antibodies, found in camelids such as llamas and alpacas, exhibit distinct characteristics from conventional antibodies due to the absence of a light chain in their structure. Furthermore, they possess a single antigen-binding domain known as VHH or Nanobody (Nb). With a small size of approximately 15 kDa, these Nbs demonstrate improved characteristics compared to conventional antibodies, including greater physicochemical stability and enhanced biodistribution, enabling them to bind inaccessible epitopes more effectively. As a result, Nbs have found numerous applications in various medical and veterinary fields, particularly in diagnostics and therapeutics. Advances in biotechnology have made the production of recombinant antibodies feasible and compatible with large-scale manufacturing. Through the construction of immune phage libraries that display VHHs and subsequent selection through biopanning, it has become possible to isolate specific Nbs targeting pharmaceutical targets of interest, such as viruses. This review describes the processes involved in nanobody production, from hyperimmunization to purification, with the aim of their application in the pharmaceutical industry.

## 1 Introduction

### 1.1 Relevance of nanobodies in medicine

Despite significant advancements in scientific and technological knowledge in the field of health, particularly in the development of biotechnology aimed at translating knowledge into practical applications, infectious diseases continue to have a substantial impact on public health and the global economic system (1). These diseases are predominantly caused by microorganisms and have been responsible for millions of deaths throughout the last century. For instance, the Spanish flu outbreak in 1918 resulted in over 50 million fatalities (2). Numerous diseases affect a significant portion of the global population.

Recently, the pandemic caused by the SARS-CoV-2 virus in 2019 has resulted in more than 6.9 million deaths as of May 2023, and it does not have an exact end date despite its slowdown (3). However, the introduction of technologies, including vaccines, antibiotics, and biopharmaceuticals, many of which have emerged with the aid of biotechnology, representing some of the most notable achievements of modern science, has contributed significantly to a substantial reduction in mortality from infectious diseases over the decades (4). Nevertheless, in Brazil, several of these infectious diseases continue to affect primarily the most vulnerable populations. For instance, between 2000 and 2015, Brazil reported a significant increase in dengue and chikungunya cases, with relevant outbreaks in major cities such as Rio de Janeiro and Salvador. Preliminary reports indicate that in 2019, approximately 132,000 cases of chikungunya and about 1.5 million probable cases of dengue in Brazil (5).

Faced with the need to control numerous diseases, coupled with an increase in life expectancy and advancements in science and technology, biopharmaceuticals have revolutionized the treatment landscape across all medical disciplines (6). These drugs, known as biopharmaceuticals, are derived from biotechnological processes that utilize cells or microorganisms for production, setting them apart from conventional drugs due to their complexity, specificity, and high efficacy. Monoclonal antibodies, hormones, and recombinant vaccines are among the most common types of biopharmaceuticals today (6–8). The potential of biopharmaceuticals is immense, driven by their high demand, as evidenced by the pharmaceutical industry, which recorded sales of approximately US $273.6 billion in 2022 (9).

An important class of biotherapeutic agents is antibodies, known for their high specificity, potency, and stability (10). The development of hybridomas in the 1970s revolutionized the production of monoclonal antibodies (mAbs), which are antibodies derived from a single B cell with specificity for a single epitope (11). Since then, mAbs have become one of the primary classes of biopharmaceuticals in the global market (7, 12). The fields of oncology and hematology have particularly benefited from these biopharmaceuticals, with the creation of 15 specific mAbs for cancer treatment and 12 mAbs for hematological diseases between 2012 and 2017 (13). However, in recent years, the pharmaceutical industry has been pursuing various targets, leading to the application of antibodies in the treatment of other diseases, including infectious diseases such as COVID-19, where antibodies have been developed and marketed (14).

Despite the significant advancements that monoclonal antibodies have brought to medicine, their primary drawbacks include high cost, technological requirements, and prolonged production times (7). Additionally, other limitations can be noted, such as elevated immunogenicity, the potential presence of contaminants in cell culture media, and challenges in scaling up production (7, 14). In this context, nanobodies, also known as VHHs, have emerged as an alternative to mAbs following their discovery in the 1990s by a group of researchers led by Raymond Hamers (15–17).

Nanobodies (Nbs) are single antigenic binding domains belonging to the heavy chain antibodies and can be found in the circulating serum of Camelids, including camels, dromedaries, llamas, and alpacas (16). These structures correspond to the smallest proteins capable of antigenic binding, with an approximate size of 15 kDa (18). Due to their small size, nanobodies offer several advantages over monoclonal antibodies, including access to epitopes inaccessible to conventional antibodies, greater stability under extreme conditions such as pH and temperature, high solubility, ease of genetic manipulation, cloning, and expression in prokaryotic systems, lower production costs, among others (17, 18).

The translational journey of nanobodies began in December 2001 with the biopharmaceutical company Ablynx^®^, which aimed to explore the therapeutic applications and production methods of these VHHs for the development of new biopharmaceuticals (19). Between 2003 and 2010, there was a significant increase in publications related to the use of Nbs as therapeutic agents, reflecting their immense potential in the field of medicine. Several patents were granted to companies worldwide during this period, including Ablynx^®^ (19). Preclinical and clinical studies investigating the use of VHHs as biopharmaceuticals and imaging agents were initiated during this time (19). In 2019, the first nanobody-based treatment called Caplacizumab^®^ was approved for acquired thrombotic thrombocytopenic purpura (TTP) (19, 20). As a result, with the relaxation of intellectual property restrictions on VHHs, numerous therapies based on these antibodies are currently undergoing clinical development.

### 1.2 Nanobodies: the smallest variant of antibodies

Conventional immunoglobulins (IgG) are complex proteins composed of two identical heavy (H) and two identical light (L) polypeptide chains that are highly conserved among mammals (21). Within this structure, the N-terminal domains of the light and heavy chains, which are responsible for antigen binding, exhibit significant variability between different antibodies. Consequently, this region is known as the variable domain (VH and VL) (21, 22). The variable region consists of the framework region (FR) and the hypervariable region, which encompasses several amino acids and governs the antibody’s specificity towards the antigen (18). Antibodies exhibit diversity in antigen recognition due to the variation in six complementary determining regions (CDRs).

**Figure 1:**
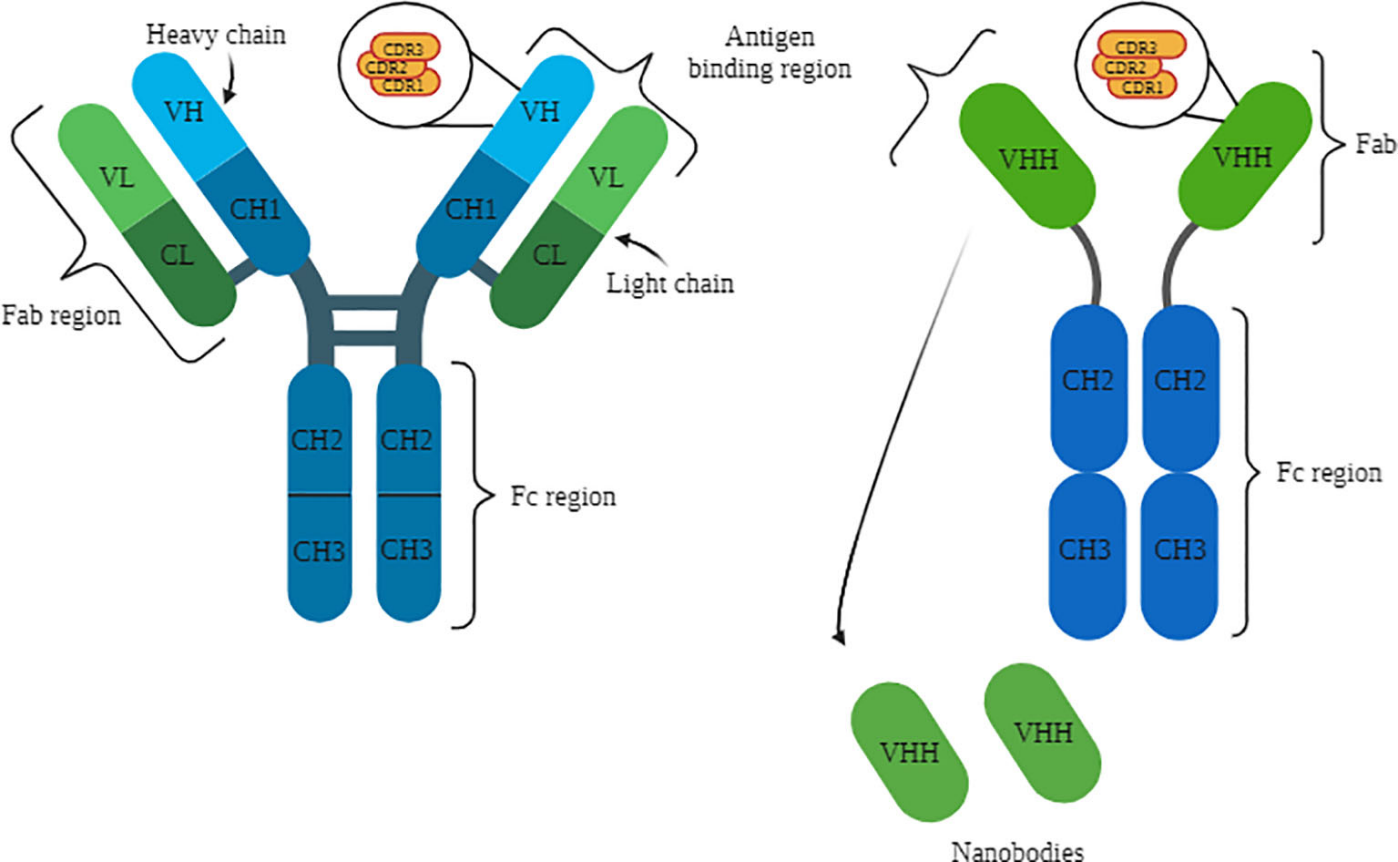
Structure of IgG on the left, with representation of the light and heavy chains, as well as the variable and constant domains, with a focus on the CDRs. HCAb structure on the right, with representation of the heavy chain and variable and constant domains. Below, the representation of the Nbs. Source: by the author, 2023. Created with BioRender.com.

The variable heavy (VH) and variable light (VL) domains together are referred to as variable fragments (Fv), while the remaining conserved structures of IgG are abbreviated as constant heavy (CH) and constant light (CL) (**Figure 1**) (21). Consequently, the paired VL and CL domains form the Fab region (fragment antigen-binding), which is responsible for antigen binding (18, 22). The remaining CH domains constitute the Fc portion of immunoglobulins, which plays a role in the recruitment of immune system cells and complement activation (21, 22). **Figure 1** illustrates the structures of conventional and camelid G immunoglobulins, highlighting their distinct domains with a specific focus on the complementary determining regions (CDRs). Camelid antibodies possess a longer CDR3 than conventional antibodies, as depicted in the **Figure 1**. Additionally, the figure also showcases the origin and structure of nanobodies.

The polypeptide chains of immunoglobulins are connected by disulfide bridges formed between cysteine residues of the light and heavy chains (22). This complex structure has an approximate size of 150 kDa and is produced by B cells in response to the recognition of pathogen proteins or polysaccharides (18). However, there are unique immunoglobulin structures that consist solely of heavy chains, with a size of approximately 90 kDa, known as heavy chain antibodies (HCAbs or heavy-chain antibodies) (18, 22).

Camelids possess both conventional immunoglobulins (IgG1) and special immunoglobulins (IgG2 and IgG3), with heavy chain antibodies (HCAbs) comprising up to 70% of these proteins in camels and up to 50% in other members of this animal group, underscoring their significant importance in their respective immune systems (18). In addition to the absence of the light chain, these antibodies lack the CH1 heavy domain, thus being composed of CH2 and CH3. These regions are connected to the single antigen-binding variable domain (VHH) through the hinge region (18, 22). Functionally, VHHs are equivalent to the Fab fragment of IgG1. However, the variable domain of HCAbs is primarily composed of hydrophilic amino acids, providing them with enhanced solubility and stability (18, 22). Furthermore, HCAbs possess a more extensive CDR3 region compared to conventional immunoglobulins, enabling them to have a larger antigen-binding surface and access regions that are typically inaccessible to IgG1 (18).

The H gene, responsible for encoding the heavy chains of conventional immunoglobulins in mammals and the heavy chain antibodies in camelids, is located at the same locus (22). This gene consists of an organized sequence of V (variable), D (diversity), and J (junction) elements that undergo somatic recombination, resulting in the formation of various antigenic recognition regions within the variable domains of the heavy chains (VH or VHH) (21, 22). During the formation of the conserved CH2-CH3 domains in HCAbs, a point mutation occurs in the nucleotide sequence of the CH1 coding exon, where a guanine is replaced by an adenine. This mutation disrupts the 5’ splicing site between the coding exons of CH1 and the hinge region, leading to the exclusion of CH1 from the mRNA sequence through splicing (22).

The monomeric antigenic binding region (VHH) is often referred to as a nanobody due to its small size of approximately 15 kDa, which corresponds solely to the variable region of HCAbs (18). This region has been extensively isolated from camelid antibody genes to produce recombinant nanobodies, with the aim of utilizing them in diagnostics and therapies (18). As a result, Nbs can be cloned and expressed in microorganisms, yielding a high production output in a simplified manner compared to mAbs (22). Moreover, VHHs can be easily modified and adapted into more complex structures to fulfill specific application requirements, such as their conjugation with the Fc portion of IgG (16).

### 1.3 Phage display as a technique for obtaining nanobodies.

For the use of nanobodies as biopharmaceuticals or diagnostic tools, it is crucial that they demonstrate specificity for the target, which corresponds to the disease to be treated or diagnosed. To achieve this, camelids are immunized with the corresponding antigen to obtain antigen-specific Nbs. These Nbs are commonly derived from immune libraries generated through selection techniques such as phage or yeast display (16, 17, 22).

One of the most common techniques for selecting proteins and antibodies is phage display (23). This technique has been widely employed due to its versatility, efficiency, and cost-effectiveness (16, 17, 24, 25). To perform this selection, the protein of interest is presented on the surface of the phage, which then interacts with a diverse range of target molecules in a process known as biopanning (25). This allows for the isolation and enrichment of specific ligands, particularly antigen-specific ligands in the case of antibodies (26).

There are two main approaches to producing recombinant phages for use in phage display. The first approach involves cloning exogenous DNA directly into the phage genome, which contains all the necessary genes for host infection, replication, and assembly (24). In this case, the use of the phage vector enables multivalent display of antibodies on the phage surface (24, 26). The second approach utilizes a phagemid vector, which combines characteristics of both plasmids and phages. This vector includes a bacterial replication origin, a selection marker with antibiotics, the gene encoding the antibody fused with the coat protein, and the phage replication origin (24, 27). However, to produce functional phages displaying antibodies, it is necessary to infect the host with a helper phage that contains all the required genes for assembly, replication, and infection (24, 26). This results in a competition between the coat protein of the helper phage and the protein synthesized by the phagemid during phage assembly (24). As a result, most resulting phages do not display the target protein on their surface, and those that do exhibit only a single copy.

Thus, in order to perform phage display, the coding gene for the protein of interest is cloned in frame with one of the viral coat protein genes (26, 27). Consequently, during host cell infection and the phage assembly process, the protein of interest is synthesized fused to the phage coat protein (26, 27). Since the genes encoding antibodies exhibit high variability due to the natural diversity of the immune system, a collection of variants known as the Library is obtained (26).

**Figure 2:**
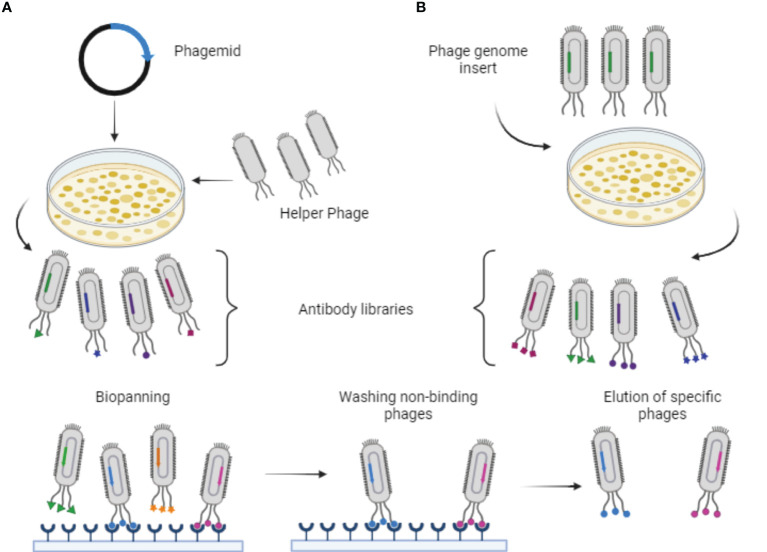
Schematic representation of phage display and biopanning processes. **(A)** Production of recombinant phages displaying antibodies using a phagemid vector and infection with helper phages. **(B)** Phages with genome modifications infecting cells to produce phages displaying the antibodies. Source: by the author, 2023. Created with BioRender.com.

**Figure 3:**
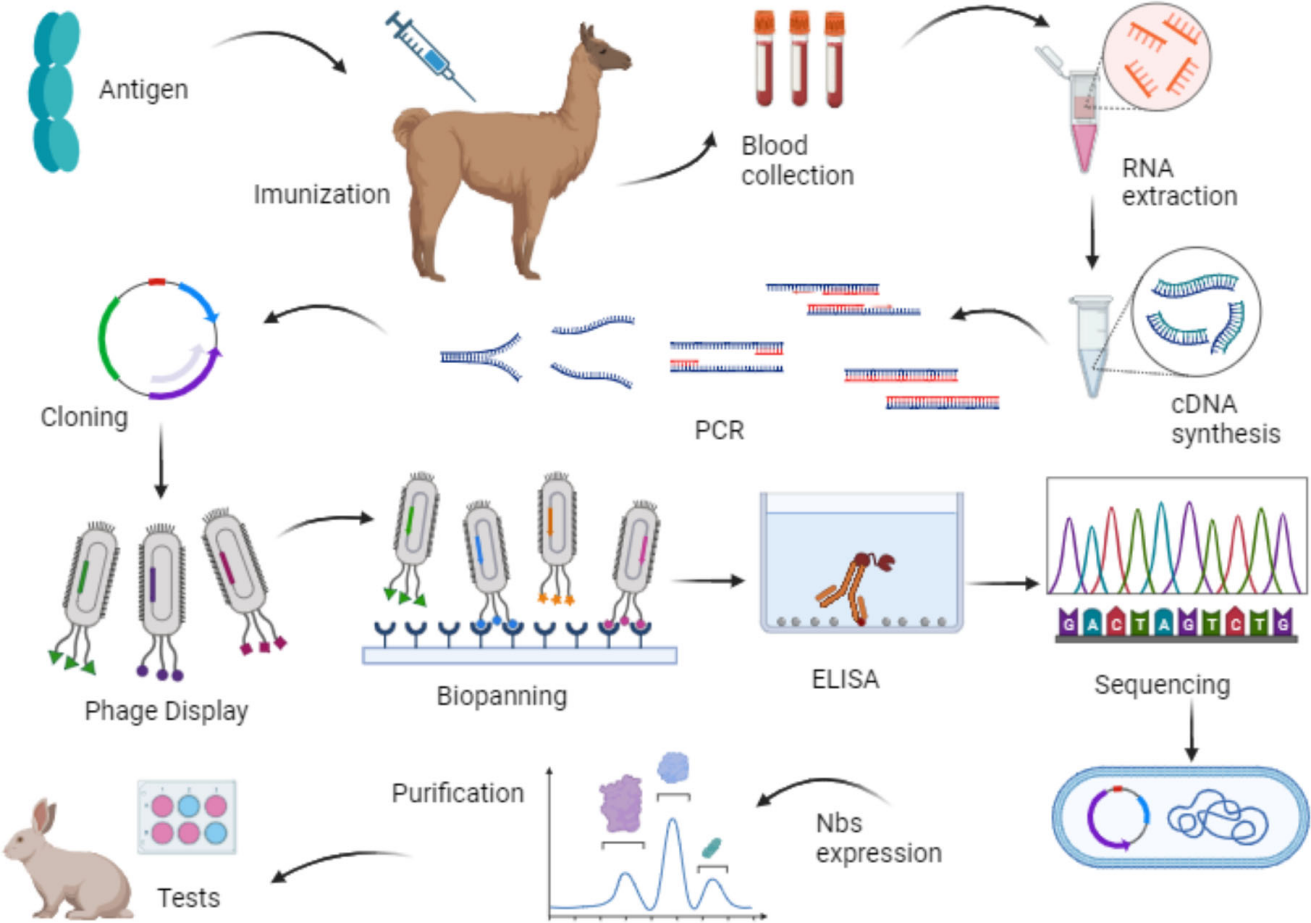
Representative scheme of the production steps of nanobodies using phage display technology. Source: by the author, 2023. Created with BioRender.com.

Then, utilizing the recombinant phage particles, each representing a clone with genetic variability, the selection of specific ligands through biopanning is conducted. Consequently, only phages that recognize the target proteins are eluted and enriched in subsequent biopanning cycles (26). This selection is based on the antibody’s affinity for the antigen, making it an excellent strategy for nanobody utilization as well (24). **Figure 2** provides a schematic representation of the Phage Display and Biopanning processes. Additionally, **Figure 3** illustrates the production of nanobodies with the intention of their application in the market. Blood is collected from the hyperimmunized animal, and the lymphocytes are isolated. From these cells, RNA is extracted and only messenger RNA (mRNA) is employed for the synthesis of complementary DNA (cDNA), which is then subjected to polymerase chain reaction (PCR) amplification. After amplifying the genetic material, the cloning process takes place in a phagemid vector or bacteriophage, responsible for translating the nanobody genes and subsequently displaying them in the phage structure. Consequently, a phage library displaying VHHs is generated. These outlined steps summarize the processes described in the selected articles and are therefore presented in a concise manner.

## 2 Objectives

The objective of this study is to present a literature review on the construction of immune libraries through immunization of camelids to produce nanobodies. The focus is to provide a comprehensive understanding of the selection technique for antigen specific Nbs using phage display, with the goal of their future application in the biopharmaceutical market.

## 3 Methods

To write the bibliographic review, we conducted a search for indexed descriptors on the DeCS (https://decs.bvsalud.org/) and MeSH (https://www.ncbi.nlm.nih.gov/mesh) platforms. **Supplementary Tables 1** and **2** provide a list of these descriptors. The search was conducted in August 2022. The selected descriptors were used to retrieve scientific articles from online databases, including PubMed, Embase, Web of Science, LILACS, Scielo, Library Cochrane, Science Direct, and CINAHL.

**Figure 4:**
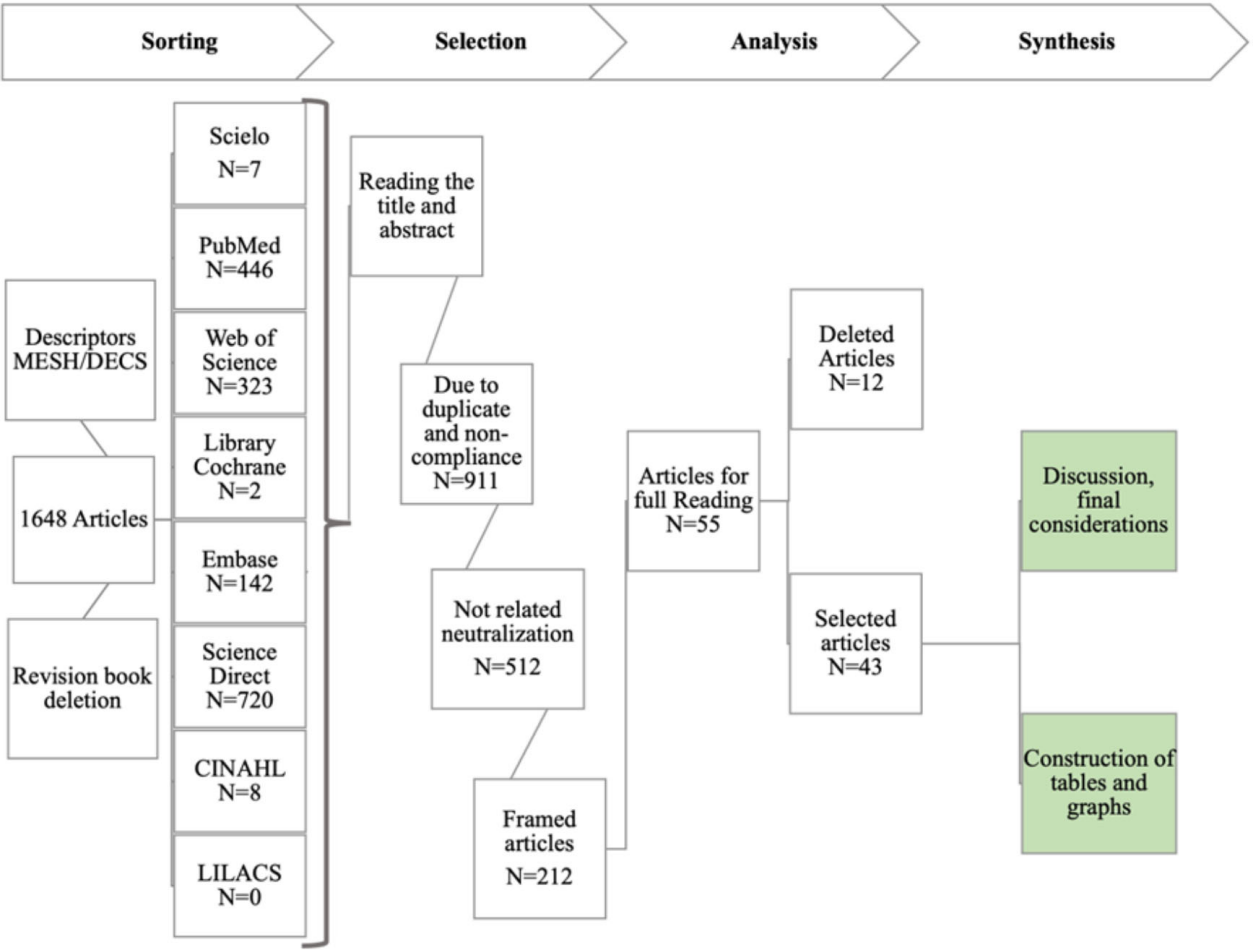
Outline of the methodology for writing the bibliographic review on: “Construction of nanobodies library from the immunization of camelids aiming to obtaining biopharmaceuticals”. Source: by the author, 2023.

The descriptors were obtained by using the Boolean operator “AND” between each line of the table and the operator “OR” between each alternative term. A temporal selection filter was also applied, including materials published between 2018 and 2022. In this manner, a total of 1,648 scientific articles were retrieved, excluding books and research reports. The number of materials and the flowchart for each online database can be found in **Figure 4**. All the articles found underwent an initial selection stage, in which the titles and abstracts were read. As a result, 911 scientific articles were excluded for two reasons: duplicates in the selection and lack of relevance to the research objectives. Another 512 articles were excluded as they did not address the production of nanobodies for viral infection. Therefore, 225 scientific articles were deemed suitable for full reading. Among these, 55 articles were selected for full reading, considering the journal in which the material was published. Additionally, 12 articles were excluded due to a lack of a clear methodology for constructing the bibliographic review. After reading the remaining 43 materials, a synthesis and cataloging of the scientific articles were performed, followed by a review with discussion and final considerations, as depicted in **Figure 4**.

## 4 Results

### 4.1 General metrics

From the full reading of the 43 selected articles, analyses were conducted on these comprehensive materials. Initially, the following aspects were evaluated: the most frequently used keywords compared to the MeSH platform descriptors, the year of publication, the number of articles related to COVID-19 and its correlation with the publication year, the list of items focusing on detection or treatment, and the list of publications focused on human or animal health.

**Figure 5:**
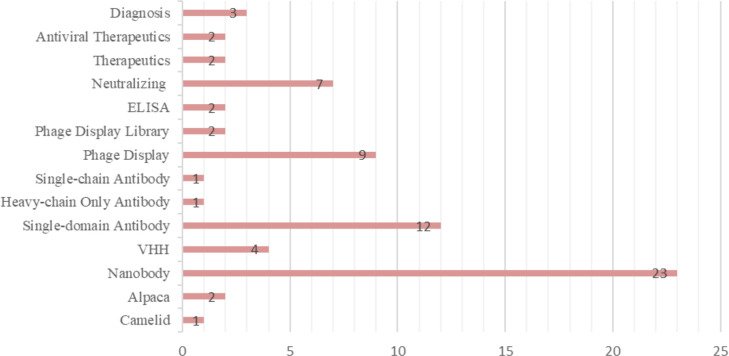
Keywords most used in selected articles.

**Figure 6:**
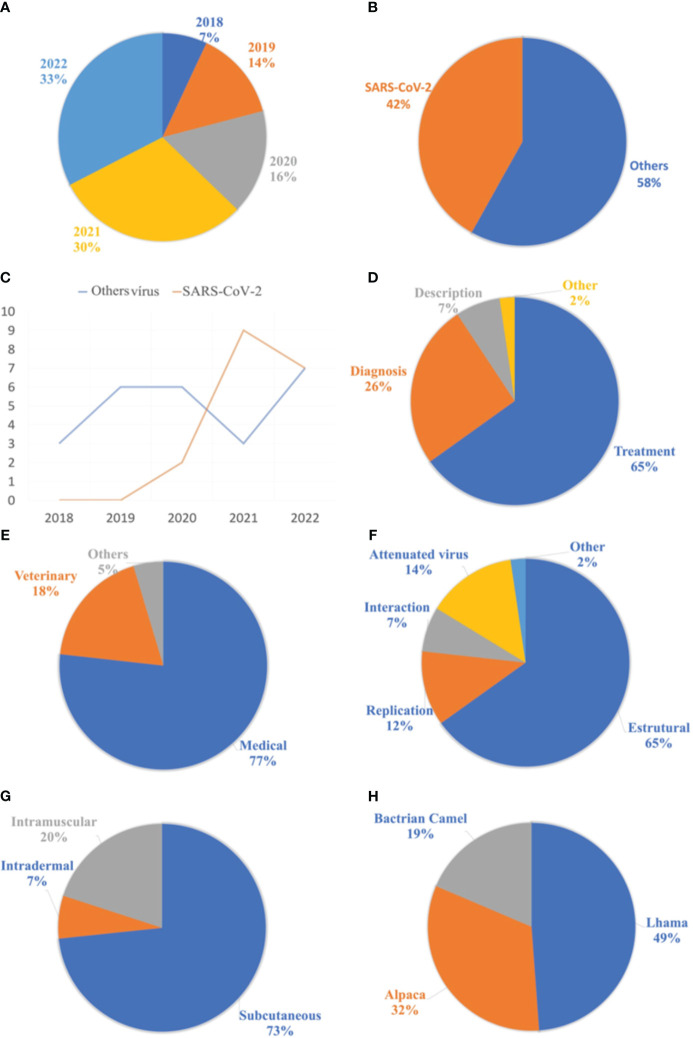
Analyzes related to publications of the nanobodies for the treatment of viral diseases. **(A)** List of publications selected by year. **(B)** On the left, list of publications between the SARS-CoV-2 virus and the various selected viruses. **(C)** list of publications between the years 2018 and 2022. **(D)** List of applications of nanobodies in selected articles. **(E)** Relationship of applications of nanobodies in different areas. **(F)** List of antigenic forms used in the camelid hyperimmunization process. **(G)** Proportion of administration routes used for antigen application. **(H)** Proportion of animals used in the production process of nanobodies, from the hyperimmunization of camelids.

The most frequently used keywords were listed and grouped into different categories based on the descriptors. The terms “Nanobody,” “Single-domain Antibody,” “Phage Display,” and “Neutralizing” were the most used. **Figure 5** presents the 14 keywords that appeared most frequently in the selected materials. Additionally, the publications were filtered by year, starting from 2018 until the retrieval date in August 2022. As a result, 3 articles were published in 2018, 6 in 2019, 7 in 2020, 13 in 2021, and finally, 14 articles in 2022. The respective percentages for each year are shown in **Figure 6A**. Among the selected articles, 42% focused on the production of nanobodies against SARS-CoV-2 compared to other viruses (**Figure 6B**). These articles were also analyzed in terms of their publication year (**Figure 6C**). A significant increase in publications was observed between 2020 and 2021, with a slight decrease in 2022. Regarding other viruses, there was a 50% decrease in the number of publications between 2020 and 2021, followed by a relative increase in 2022. Due to the high stability and specificity of Nbs, they can be applied both in the treatment of infectious diseases and in the detection of these agents (**Figure 6D**). Therefore, the percentages of articles selected for each application of these antibodies were analyzed. In the selection process, 65% of the studies focused on disease treatment, 26% on diagnosis, and 7% only provided characterization of the nanobodies. One selected publication deviated from the categories, as it used VHH as a tool for adenovirus purification in chromatographic processes, representing 2% of the publications (28).

The market for nanobodies extends beyond their application in medicine and human health, as they can also be utilized in veterinary medicine, biotechnology, and even agronomy (**Figure 6E**). Accordingly, a list of nanobody applications in the selected materials was compiled, with 77% of the publications focusing on human health and 18% on animal health. The remaining publications categorized as “others” refer to two articles: the first utilizing Nb as a tool in adenovirus purification (28), and the second employing this antibody for the detection of viruses that cause disease in plants (29).

### 4.2 Immunization of camelids

Starting from the selected articles, information regarding the first phase of nanobody production was compiled and organized into tables. This initial stage includes details such as the infectious agent, the antigenic form used for immunization, the adjuvants, the route of administration, the animal species, the number of immunizations, and the antigen dosage.

When analyzing the antigenic forms used for immunizing camelids, only 14% of the studies employed attenuated or inactivated virus vaccines. The majority used recombinant proteins during the hyperimmunization process. Viral proteins can be classified into structural and non-structural categories, further divided into replication and interaction proteins (30). Among these studies, 65% utilized structural proteins in the hyperimmunization process, as they were considered highly immunogenic. The distribution of recombinant viral proteins is presented in **Figure 6F**.

Regarding the adjuvants used in the hyperimmunization process, 37% employed complete Freund’s adjuvant for the initial application, followed by incomplete Freund’s adjuvant for subsequent applications. The second most frequently used adjuvant was Gerbu^®^, accounting for 7% of the selection. Aluminum hydroxide and Poly (I:C) adjuvants were also used, representing 5% and 2%, respectively. However, 16% of the studies did not report which adjuvant was used. Among the selected materials that provided information on the route of antigen administration, the subcutaneous route was widely employed, representing 73%. Other routes, such as intramuscular and intradermal, were also mentioned, accounting for 20% and 7%, respectively (**Figure 6G**).

Regarding the animal species naturally producing nanobodies, llamas, alpacas, guanacos, camels, and dromedaries stand out. Analyzing the animals selected for nanobody production from immune libraries, only three species were utilized, with llamas accounting for 49% of the total. Alpacas were the second most used species in this process, representing 32%, followed by Bactrian camels with 19% (**Figure 6H**). Guanacos and dromedaries have greater resistance to domestication and therefore are not commonly used in nanobody production (31).

**Supplementary Table 4** presents the compiled data, with viruses grouped into families for comparison purposes. The main information regarding the hyperimmunization process of camelids is summarized, including the antigenic form used, adjuvants, route of administration, animal species, interval between immunizations, and antigen dosage.

### 4.3 Construction of the phage library

In the second stage of nanobody production, the construction of the phage library is performed, displaying the VHHs in its structure (**Figure 3**). **Supplementary Table 5** presents the information used in the selected materials regarding the construction of the phage library.

After collecting blood from the hyperimmunized animal, lymphocytes must be isolated. The most used approach, in the selected materials, was density gradient centrifugation. In this approach, peripheral blood mononuclear cells, including lymphocytes, are separated based on their densities in relation to the solvent used. Following cell isolation, RNA is extracted. Various methods are used for RNA extraction, with commercial kits being the most employed. Among these kits, TRIzol^®^ is highlighted. However, some authors performed RNA extraction using phenol-chloroform. During the extraction, total RNA or mRNA can be obtained from the cells, depending on the technique and materials employed.

The next step is the synthesis of cDNA through reverse transcription, which requires primers to bind to the RNA and synthesize cDNA using the enzyme reverse transcriptase. The most used primers by the authors were oligo d(T), which have a thymine tail responsible for binding to the poly A tail of mRNAs. This primer allows for the selection of only the mRNAs from the total RNA pool extracted, which is essential in the expression of recombinant proteins in prokaryotic systems. Random primers were also used, consisting of a mixture of oligonucleotides representing all possible sequences for binding to RNA, resulting in cDNA of varying lengths. The least commonly used primers in the selected materials were specific gene primers, which bind to the RNA in the target region. Many authors combined oligo d(T) and random primers to improve efficiency and transcript representation in the reverse transcription process.

**Figure 7:**
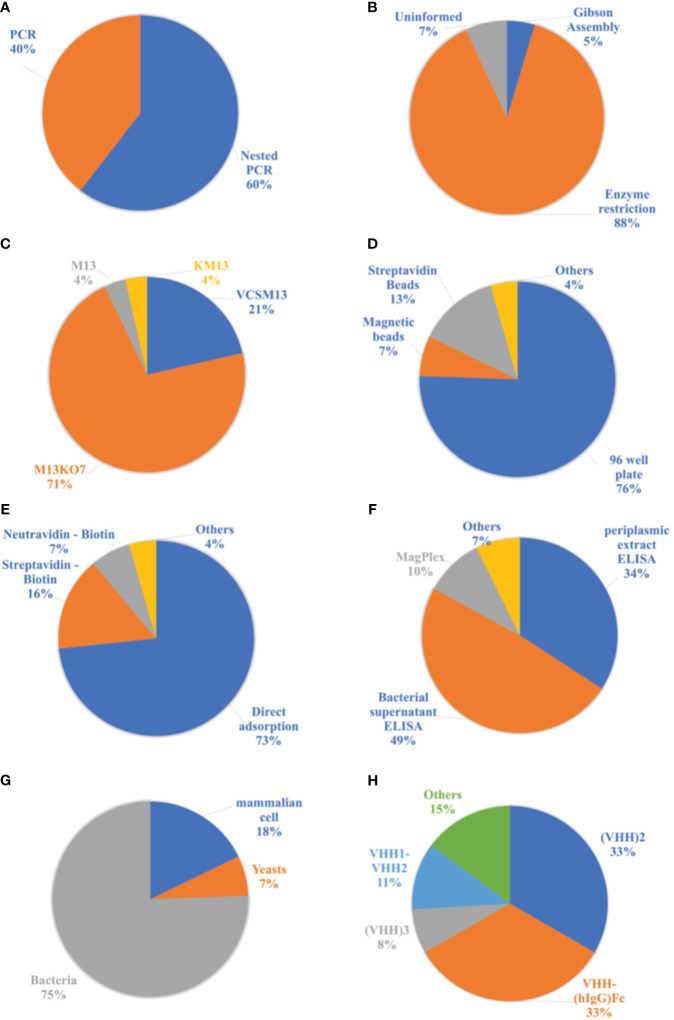
Analyzes related to publications of the nanobodies methodologies. **(A)** Proportion of amplification reactions of genetic material used in the production process of nanobodies. **(B)** List of cloning processes used in the production of Nbs. **(C)** List of helper phages used in the cloning process with a phagemid vector. **(D)** List of supports used for displaying antigens in the biopanning process. **(E)** List of antigen immobilization methods for biopanning. **(F)** Ligand detection methods used to select Nbs. **(G)** Cells used in the nanobodies expression process. **(H)** Most frequent modifications of Nbs in the selected literature.

In the next step, the target genetic material is amplified from the synthesized cDNA through PCR or Nested PCR. In this process, primers specific to the outermost regions of the DNA are initially used, targeting the CH2 region in the case of HCAbs. In the first reaction, fragments of approximately 700 base pairs (bp) corresponding to HCAbs (VHH-CH2) and 900 bp corresponding to conventional IgG (VH-CH1-CH2) are obtained. Subsequently, agarose gel electrophoresis is performed, followed by extraction and purification of the 700 bp fragments. These genes are then used in a second PCR reaction, specifically amplifying the VHH genes to obtain fragments of approximately 450 bp. Among the analyzed publications, 60% of the authors used a double amplification process known as Nested PCR. However, 40% of the authors employed conventional PCR for the amplification of the VHH genes (**Figure 7A**).

The amplified genetic material of interest is then cloned into a phagemid vector or a bacteriophage. Among the analyzed materials, only one study used bacteriophages, specifically T7 phages as vectors. The remaining studies utilized a plasmid vector due to its ease of application, with pHEN, pMECS, pECAN, and pCOMB vectors being prominently used. Cloning involves the insertion of DNA fragments into the vector. Among the selected publications, 88% used the restriction enzyme cloning process, which employs a restriction enzyme to create cohesive ends in the DNA fragments and vector. After digestion with enzymes, these materials are ligated through intermolecular interactions and the action of binding enzymes, resulting in the insertion of the DNA sequence into the vector. However, 5% of the selected articles employed a process called Gibson Assembly, which does not rely on restriction enzymes and involves the overlapping of DNA fragments between the gene and the vector. The cloning method used in other publications could not be identified (**Figure 7B**).

The cells most used in the cloning process with the phagemid vector were transformed through either electroporation or thermal shock to incorporate the vector. Bacteriophages, on the other hand, have the natural ability to infect cells and transmit the target along with their genetic material. The *E. coli* TG1 bacterial strain was the most frequently used in this process, followed by the *E. coli* XL1 Blue strain.

To use the phagemid vector in the cloning process, it is necessary to perform superinfection of the transformed cells with a helper phage. This infection generates functional phages capable of replicating and infecting new cells. Thus, the phage library displaying the VHHs is produced. Among the authors who used the phagemid vector, 71% employed the helper phage M13KO7 in the superinfection process. The VCSM13 phage was the second most used, accounting for 21%. The M13 and KM13 phages were also used, but to a lesser extent (**Figure 7C**).

**Supplementary Table 5** presents the compiled data from the selected articles regarding the construction of the phage library displaying VHHs. The essential steps in the process of building the library through hyperimmunization of camelids are summarized. Thus, RNA extraction, cDNA synthesis, amplification of genetic material, cloning, and the phage display process are described in **Supplementary Table 5**.

### 4.4 Isolation of nanobodies

The third stage of producing nanobodies consists of selecting and identifying those with the best characteristics, such as the highest affinity and the lowest cross-reaction. This is achieved through an initial enrichment step performed by biopanning, where the antigens are exposed and interact with the phages displaying the VHHs. After a non-specific phage washing process, the bound phages are eluted and used to infect new cells for a second round of biopanning. The method of detecting positive binders is applied to the enriched phages displaying Nbs with target specificity. In this method, the antibodies that present a more intense positive signal are selected, followed by sequencing and expression.

From the library of phages displaying VHHs obtained in the second stage, phages are selected based on their specificity to the target. The VHHs can either bind or not bind to the antigen immobilized on solid supports for display. The most used supports in this process are 96-well plates (microplates) and beads. Among the selected materials, 76% used 96-well plates, while 13% used streptavidin beads and 7% used magnetic beads. The immobilization of antigens in agarose beads and immunotubes was also mentioned, classified as “Others” in **Figure 7D**.

For the immobilization of antigens on the supports, the most used methods are direct adsorption, where the antigen is incubated with the support, or the use of proteins such as streptavidin and neutravidin. When using these proteins for immobilization, it is important for the antigen to have biotin in its structure, enabling interaction with these molecules. In the latter strategy, the antigen is biotinylated prior to biopanning. Microplates can be coated with streptavidin or neutravidin, and beads can also contain these proteins in their composition, such as Dynabeads^®^. Among the antigens used, 73% were immobilized through direct adsorption on plates, magnetic beads, agarose beads, or immunotubes. However, 16% used streptavidin for immobilization on plates or beads, and 7% used neutravidin only on plates. The remaining 4% employed unconventional forms of immobilization, such as maltose-binding protein and IgG from immunized llamas (**Figure 7E**).

The number of biopanning cycles for selecting phages displaying antigen-specific Nbs may vary from study to study. The number of cycles ranges from one to four, with two and three repetitions being the most common. Among the selected articles, 17 performed two cycles of biopanning for phage enrichment, while a total of 20 articles performed three cycles. **Supplementary Table 3** below provides the number of articles and the number of cycles performed during biopanning.

After the enrichment process, the ability of nanobodies to bind to antigens is tested. Usually, ELISA assays (enzyme-linked immunosorbent assay) are the most used method, and the substrate analyzed may vary. However, magnetic beads such as MagPlex^®^ have also been applied in binder detection assays. In this context, 83% of the publications determined the nanobodies capable of binding to the antigen through ELISA assays. However, 34% of the total used the periplasmic extract after inducing the expression of the nanobodies, meaning that the soluble Nbs in this medium interacted directly with the antigens. Furthermore, 49% of the materials used the bacterial supernatant, where the Nbs displayed by the phages interacted with the antigens. This technique is commonly referred to as phage ELISA. Magnetic beads were used in 10% of the selected materials. Other methods, such as Western Blotting and immunofluorescence studies on transfected HeLa cells, were also employed in the detection of binding Nbs, as depicted in **Figure 7F**.

Subsequently, the vectors used to build the phage libraries, which showed a positive signal in the previous tests, are sequenced. Sequencing is mostly performed using NGS, but some studies have employed the Sanger method. The determined sequences are then grouped into families based on the length of the CDR3. **Supplementary Table 6** summarizes the selection process of Nbs ligands using the phage display and biopanning steps. The supports used in biopanning are described, including the method of antigen immunization on the support, the amount of these proteins used, the number of cycles performed, the method of detecting the ligands, and the number of families obtained in each study.

### 4.5 Obtaining and testing nanobodies

The fourth stage in the production of nanobodies, based on the construction of immune libraries, involves the expression of recombinant proteins, followed by their purification. Purified Nbs undergo various characterization steps and may be tested for their neutralizing potential, depending on the purpose of their production. Initially, the phage display vector is subcloned into an expression vector, or alternatively, the transformed cell can be changed while keeping the same vector. There are several commercially available options for expression vectors, phage display systems, and cells that can be used in this process. Combining different options is also feasible and commonly practiced. Subsequently, the transformed cell is cultured until an ideal time for inducing expression. The expressed nanobodies are harvested and purified, typically through chromatographic processes. After purification, they are characterized for their affinities and structures. Nbs intended for the treatment or prophylaxis of diseases are subjected to neutralization assays.

Among the selected articles, 30% used the phagemid vector, which is employed in the phage display steps, for the expression of nanobodies, only replacing the bacterial strain with a non-suppressor strain of the amber stop codon. In this process, the phage pIII protein is not produced, and only VHH is expressed. However, 70% of the publications utilized a new expression vector and a compatible cell line. The most widely used vector was pET22b in a prokaryotic expression system with *E. coli* DE3. Due to the simplicity and practicality of microbial cultivation, especially with *E. coli*, 75% of the selected materials employed bacterial cells for nanobody expression. However, yeast cells accounted for only 7% of the publications, with *Pichia pastoris* being the most frequently used. Mammalian cells pose greater challenges in terms of cultivation, but they were also utilized for nanobody production, representing 18% of the total number of selected publications, as shown in **Figure 7G**.

After the production of recombinant nanobodies, purification is performed to subsequently analyze the structure and neutralizing capacity of these antibodies. Chromatographic processes are the most frequently used for purification, both on a small and large scale. However, column systems in tubes (spin columns) have also been employed for the purification of these antibodies on a small scale. The choice of purification method depends on the physical and chemical characteristics of the nanobodies, as well as the final objective of their production. The processes of affinity chromatography with immobilized metal ions (IMAC) and size exclusion chromatography were the most prominent and often combined during purification. Since most plasmids add a Histidine (His-tag) or Hemagglutinin (HA-tag) tail to the recombinant protein, the most frequently mentioned columns were HisTrap^®^, Ni-NTA, and Ni-Sepharose. These columns retain the nanobodies in their resins through intermolecular interactions. Imidazole is commonly used for eluting the Nbs at the end of the IMAC chromatographic process.

After purification, the VHHs are characterized in order to evaluate their structure, avidity, stability, epitope recognition, and other characteristics, which vary in each study. Among the characterization tests are ELISA, Bilayer Interferometry (BLI), surface plasmon resonance (SPR), Cryo-electron microscopy, circular dichroism, and others (**Supplementary Table 7**).

Varying according to the different applications of these antibodies, some modifications were made to their structure, such as their conjugation with the Fc portion of human immunoglobulin G (VHH-(hIgG)Fc) or the construction of dimers. Thus, 44% of the studies made modifications, and these constructs were applied in the treatment and prophylaxis of diseases. By implementing these modifications, improvements in the efficiency and avidity of the Nbs can be achieved, in addition to increasing the half-life of these antibodies in the bloodstream. **Figure 7H** illustrates the most common modifications made. Dimeric nanobodies were the most frequently constructed, with 33% being homobivalent (VHH2) and 11% heterobivalent (VHH1-VHH2). VHH-(hIgG)Fc accounted for 33% of the literature, and nanobody trimers (VHH3) accounted for 8%. Additionally, other modifications have been reported, including tetrameric nanobodies (VHH4) and dimers of nanobodies conjugated to the Fc portion of human IgG ((VHH)2 - (hIgG)Fc), which can be homo or heterobivalent. These modifications are represented in **Figure 7H** under the “Others” category.

Materials intended for the treatment and prophylaxis of viral diseases must undergo neutralization tests, which guide their applications in clinical trials. However, publications focused on diagnosis did not perform neutralization tests. Thus, initial plaque neutralization tests are carried out using cell cultures. In this test, infection of cultured cells will not occur if the nanobodies are able to successfully neutralize the viral particles. Flow cytometry assays are usually performed next, identifying surface molecules indicative of viral infection. The real-time PCR technique is also widely used to detect viral load in cells. Finally, *in vivo* tests are conducted in different animals to analyze the neutralizing capacity, avidity, and potency of these antibodies. **Supplementary Table 7** summarizes the process for obtaining and testing the previously selected binding Nbs. It includes the expression vectors employed in this process, the transformed cells, the method of Nb purification, descriptions of the modifications made to the antibodies after their expression, the characterization assays, and the neutralization tests conducted.

## 5 Discussion

### 5.1 Immunization of camelids

Camelids are incredibly important mammals for society, particularly for the Andean peoples. This family of animals includes llamas, alpacas, guanacos, vicuñas, camels, and dromedaries (31). All these animals naturally produce heavy chain antibodies. However, guanacos, vicuñas, and dromedaries are wild animals and therefore not utilized in the production of nanobodies for biopharmaceutical development (31). Llamas were the most used species in the selected studies, likely due to their wider territorial distribution, ease of handling, and docility.

To obtain nanobodies from immune libraries, camelid immunization is necessary. Various strategies have been employed in this process. The administration of recombinant antigens was the most frequently used strategy, accounting for 84% of the materials. This approach has gained prominence with the advancement of biotechnology as it allows to produce immunodominant epitopes in prokaryotic systems, which are highly productive. These epitopes are stable, induce a more specific and potent immune response, and are generally conserved regions in the evolutionary process (32). Consequently, they have become prominent in the hyperimmunization process. However, the immunodominant regions are not always known for the target antigen, or they can generate a negative bias in the humoral immune response, limiting the recognition of certain functionally important epitopes (33). Therefore, another strategy employed in the hyperimmunization process is the administration of attenuated virus, which eliminates the need to know the immunodominant epitope and can generate an immune response to different regions of the antigen. However, only 14% of the materials used attenuated virus or attenuated virus vaccine during immunizations.

Furthermore, the use of adjuvants in antigen administration also impacts the immune response of the animal. Adjuvants are commonly used to ensure a stronger response, especially in immunization processes using highly purified proteins, such as recombinant proteins (34). However, the route of administration, the interval between applications, and the amount of antigen used can also influence the immune response of the animal. Nevertheless, obtaining a high titer of functional and protective antibodies after immunization is crucial to produce nanobodies in the field of medicine. Therefore, recognizing the aforementioned factors is extremely important.

### 5.2 Construction of the immune library

The phage display technique for antibody generation is a dynamic process, requiring strategic choices to obtain antibodies with high specificity and sensitivity. Rigor in selecting the steps and methods involved in this technique, considering the specific nuances of each, is crucial for obtaining antibodies with the desired characteristics. Therefore, the phage display technique demands extreme attention to the specific details of each step, such as the chosen phage display vector, molecular techniques used for generating the V gene repertoire, methods of antigen immobilization, blocking agents, and elution during biopanning, among others. Consequently, different methods and strategies are employed to produce high quality libraries, each presenting specific advantages and disadvantages (23).

Lymphocytes are cells of the immune system capable of specifically recognizing foreign antigens and generating a biochemical response. They are considered the mediators of humoral and cellular immunity (21). Antigens are recognized by distinct subpopulations of B lymphocytes and T lymphocytes. However, only B lymphocytes have the ability to produce antibodies (21). Therefore, for the construction process of the immune library, the genetic material responsible for encoding the nanobodies must be isolated, specifically the material from B lymphocytes.

The blood of the immunized animal is collected in tubes containing anticoagulant substances such as EDTA or heparin. These substances prevent coagulation and allow the lymphocytes to remain in the cellular fraction of the blood, enabling their isolation (35). The next step involves gradient centrifugation, where the peripheral blood mononuclear cells (PBMCs) are separated. During centrifugation, different layers containing different cell types are formed based on their density (35). PBMCs are found in the buffy coat, and since they are the main source of lymphocytes, they are commonly used for isolation.

To produce recombinant nanobodies, cloning and expression are necessary, often in prokaryotic systems. Therefore, it is crucial to use mRNA as a template in cDNA synthesis. mRNA only contains coding regions, which are necessary for producing functional proteins. Since prokaryotes do not undergo the splicing process, using DNA as a template would result in non-functional proteins because non-coding regions of the DNA are not removed in these cells. Therefore, mRNA is extracted from B lymphocytes to initiate the library building process. Commercial kits, such as TRIzol^®^, were commonly used for mRNA extraction. Oligo d(T) and random primers are used to separate the mRNAs from the total RNA pool and synthesize cDNA with the help of reverse transcriptase. Alternatively, separation of mRNA from the total RNA pool and cDNA synthesis with gene-specific primers can also be performed, although these methods have been less reported.

The synthesized cDNA is then amplified by PCR. Various approaches can be employed during the amplification of genetic material. Nested PCR was the most used technique. This technique allows for increased specificity by sequentially amplifying fragments using different pairs of primers (36). Initially, gene fragments corresponding to IgG are amplified with specific primers, resulting in amplicons of different sizes corresponding to conventional IgG and heavy chain antibodies. In the agarose gel electrophoresis process, the smaller fragments corresponding to HCAbs are purified from the gel and used as templates in the second amplification. Specific primers for VHHs are used in the second stage to obtain them with higher specificity. The most cited primers in sequential amplification by Nested PCR were CALL001, which targets a conserved region of the variable domains, and CALL002, which targets a highly conserved region of the CH2 domain among IgG isotypes (37). After separating the fragments generated by these primers, the 700 bp band corresponding to HCAbs is selected. These fragments, along with the VHH-Back and VHH-For primers that anneal to the heavy chain variable domain, are used to produce 450 bp fragments.

For the construction of the phage library displaying VHHs, it is initially necessary to obtain a repertoire of V genes from the variable binding region of immunoglobulins, which was entirely derived from B lymphocytes of camelid PBMCs. The amplification of these genes was predominantly performed through Nested PCR, as it can provide greater specificity in amplifying VHH genes. This is particularly relevant since camels also express conventional immunoglobulins, and thus, Nested PCR allows for the selective amplification of only the genes from the heavy variable domain.

However, amplifications of VHHs by conventional PCR, starting from previously synthesized cDNA, have also been reported. In these reactions, specific pairs of primers targeting the heavy chain variable domain were used, directly obtaining 450 bp fragments.

After obtaining the VHHs, the process of cloning in a phagemid vector or bacteriophages is carried out for the phage display stage. The restriction enzymatic cloning process, known for its simplicity, was commonly used in most of the selected materials to insert fragments into the vector. For this purpose, the VHH-specific primers need to contain the recognition sequence for the restriction enzyme. The vector itself intrinsically contains these recognition regions. When both the primers and the vector are digested with restriction enzymes, sticky ends are formed, allowing them to be joined together through intermolecular interactions and the action of binding enzymes like T4 DNA ligase. This enables the insertion of the DNA fragment into the vector.

Phagemid vectors were more commonly used in the phage display process compared to bacteriophages, primarily due to the simplicity of using plasmids and compatible bacterial strains. However, when using these plasmids, the addition of helper phages becomes necessary to optimize the assembly processes of phages displaying VHHs, their replication, and host cell infection. Currently, there are several commercially available helper phages, but M13KO7 and VCSM13 were the most frequently used. Both are derived from filamentous phages but have some genetic differences between them. M13KO7 is a classic helper phage and one of the first to be used in the phage display process. On the other hand, VCSM13 was optimized to promote better display of proteins and cell infection (38, 39).

The choice of phage display vector is a crucial variable in the final outcome of the selection process. Opting for the use of the complete bacteriophage as a vector or specific Phagemid vectors, such as pJB12 combined with the M13 K07ΔpIII hyperphage system (40), will result in the multivalent display of VHHs on their coat proteins. Some authors emphasize the possibility of spatial interferences caused by the multivalent display of proteins during phage interaction with their host, depending on the size of the displayed peptide. This phenomenon may exert an impact on the infectivity of the virus, thereby influencing the diversity of the library (41, 42). Additionally, using the complete bacteriophage as a vector may cause deleterious effects during the cloning of large DNA fragments into the phage genome (41, 42). However, Phagemid vectors can display only one copy of antibody fragments conjugated to the pIII protein. This facilitates the selection of antibodies with higher affinity, avoiding avidity effects during biopanning. In other words, this approach allows eliminating the selection of clones with apparent high affinity, caused by the simultaneous binding of multiple ligands to a target (42, 43). Multivalent interaction can mask the affinity of each antibody, giving a false impression of high affinity (43).

Currently, the prevailing practice involves the use of phagemid vectors, wherein the monovalent display of proteins fused to the phage protein occurs. This is evidenced by the fact that only one study opted to employ the entire bacteriophage (T7 Select^®^) (40), and only one utilized the Phagemid vector pJB12 in combination with the M13 K07ΔpIII hyperphage system for the multivalent display of VHHs (40).

Also, the selection of the display platform employed by different research groups plays a significant role in the phage display technique for antibody generation, as it can affect expression in phages, yeast, mammalian cells, or other platforms (23). This choice is intrinsically linked to the specific objectives of each group. Expression in bacteria is often an initial step, but if a suitable binder for an antigen cannot be identified, other expression systems may be explored. Systems such as yeast and mammalian cells prioritize post-translational modifications, such as glycosylation, and offer alternative antibody formats that may enhance affinity for the target.

The quality of the antibody library plays a pivotal role in determining the final quality of isolated antibodies throughout the selection process. There is a need for these antibodies to accurately mirror the immunological repertoire, demonstrating affinity and specificity for the desired target. One of the characteristics directly influencing the quality of the library is its size, as larger libraries increase the likelihood of obtaining antibodies capable of recognizing the target (42, 44). Additionally, the diversity of the library also impacts the selection process, as it is responsible for generating a broad spectrum of antibodies capable of effectively binding to specific targets (44). Another relevant aspect to consider is the affinity of the antibodies obtained at the end of the campaign, a crucial feature in the development of molecules with therapeutic properties. This feature stands out when analyzing the library’s quality, as a positive correlation has been observed between the affinity that the obtained antibodies can achieve and the size of the library. In other words, a larger library provides the potential to identify antibodies with higher affinity (42). It is worth noting that immune libraries generally have the ability to generate antibodies with superior affinities compared to Naive and artificially synthesized libraries, although there is less diversity, as the immune library has already been directed towards a specific target through the immunization process. In this scenario, the unique characteristics of Nbs make them excellent candidates for constructing antibody fragment libraries. Due to their reduced size, the processes of amplifying the variable heavy domain, cloning, and recombinant expression are facilitated. Moreover, numerous studies have reported Nbs with high specificity and affinity for a variety of targets, along with excellent developability characteristics (44).

Few studies directly compare different established antibody formats, such as single-chain variable fragment (scFv), antigen-binding fragment (Fab), and VHH (nanobodies). However, it is important to note that, when available, these studies provide valuable insights into the distinct characteristics and relative performance of these formats. Regarding the developability of different antibody fragments, it is noteworthy that scFv and Fab exhibit approximately two to three times the molecular weight of Nbs, respectively. The compact size of nanobodies is highly relevant in applications requiring high tissue penetration. However, this characteristic can be detrimental due to their molecular weight falling below the glomerular filtration limit (45). To overcome potential limitations, alternatives have emerged, such as humanization and conjugation of Nbs. These approaches aim to address high clearance rates, prolonging the half-life of the antibody fragment in circulation and enhancing avidity by constructing dimers and trimers of VHH (45).

The reduced size of Nbs not only facilitates their genetic manipulation compared to scFv but also simplifies the construction of the phage library. Only one RT-PCR reaction is capable of providing gene fragments to build the library, whereas for scFv, multiple RT-PCR reactions are required to amplify the VH and VL genes, as well as to connect these fragments, which is typically a challenge due to the low efficiency of the process (45).

Nanobodies (VHHs) exhibit a reduction of three complementarity-determining regions (CDRs) compared to human IgG and its fragments for antigen interaction. The presence of an extended CDR3 suggests a likely adaptation to this characteristic, enabling heavy-chain antibodies to bind to a broad range of antigens often inaccessible to conventional antibodies (46). Additionally, upon binding to an antigen, VHHs demonstrate a greater stability gain, resulting in a more robust complex with the antigen compared to the already stable complex formed by VH-VL (45, 46).

The increased solubility of nanobodies compared to different antibody fragments is attributed to the replacement of four highly conserved amino acids with more hydrophilic ones (18). In the case of scFv, hydrophobic residues assist in the binding between VH and VL, resulting in lower solubility (45). As a consequence of low solubility, aggregate formation poses a significant challenge, especially for the recombinant expression of these fragments (45, 47). Furthermore, the aggregation of these proteins presents a risk of increased immunogenicity and a decrease in their biological function (47).

Minimizing immunogenicity caused by biological products represents one of the major challenges faced by biopharmaceuticals. The initial murine-derived monoclonal antibodies (mAbs) were associated with considerable immunogenicity attributed to the intrinsic presence of exogenous structures. With the development of chimeric and humanized mAbs, there has been a significant reduction in the immunogenicity of these biopharmaceuticals. This reduction is attributed to the exchange of the murine Fc portion for the human Fc, which inherently exhibits much lower immunogenicity (48, 49). Additionally, the less immunogenic variable domains have contributed to this reduction (48). However, murine variable domains still exhibit approximately 50% homology with those of humans, potentially causing undesired reactions. In contrast, VHHs show high similarity (75-90%) in sequences with the human VH domain, further potentially reducing immunogenic reactions (45, 49). Furthermore, antibodies with conventional structures may induce the production of anti-idiotypic antibodies in some patients, as well as anti-murine antibodies (48, 49).

Thus, the ongoing search for new antibody formats plays a crucial role in advancing immunotherapies, primarily due to the possibility of significantly enhancing therapeutic efficacy with the development of these antibodies. However, the diversity of antibody formats allows for a more comprehensive, synergistic, and personalized therapeutic approach, adapting to the individual complexities of each patient (49). Therefore, new formats, such as nanobodies, offer the advantage of being more easily humanized, reducing undesired immune responses, and promoting safer and more enduring therapies. The choice between these formats may depend on intended applications and desired antibody characteristics, such as tissue penetration, production capacity, and ease of genetic engineering. While the literature on direct comparisons may not be extensive, the growing research on VHH nanobodies highlights their potential and advantages, positioning them as a promising option compared to conventional antibody formats.

### 5.3 Isolation of nanobodies

A critical step in generating antibodies through phage display for high specificity, in addition to library size and diversity, is biopanning. There are different approaches to selecting specific binders, but the choice among them depends on various factors such as the purity and biochemistry of the antigen used in the selection, the immobilization method and its concentration, the support used, as well as the washing and elution conditions. These factors directly influence the quality of Nbs displayed by phages at the end of the biopanning cycle. These phages will be used in subsequent amplifications for the enrichment of binding phages.

For the isolation of nanobodies, the phage enrichment process by biopanning is initially carried out. In this process, phages displaying VHHs with greater target specificity are selected. Two different methods are commonly used for antigen immobilization on solid supports: passive adsorption and biotin conjugation (50). Passive adsorption involves directly immobilizing the antigen onto the support without the addition of chemical substances that may interfere with antigen binding. However, this method can result in the antigen being immobilized in an orientation that obstructs the antigenic binding site, preventing binding to the Nbs (51). The second strategy involves biotinylating the antigen and capturing it using streptavidin molecules. This approach allows for guided antigen immobilization, preventing obstruction of the antigen binding site. However, this method incurs higher costs due to the use of immobilization molecules such as biotin and streptavidin, and there is a possibility of selecting Nbs with affinity for these immobilization molecules (51). Nonetheless, 73% of the selected materials used the direct passive adsorption immobilization process, likely to reduce costs and due to its well-established nature.

Regarding the choice of support and the method of antigen immobilization used for generating the phage library displaying VHHs, direct adsorption on microplates was the chosen method in the majority of studies. However, this direct immobilization may alter the antigen’s conformation, masking important epitopes and affecting binding efficiency, resulting in the selection of phages specific to epitopes that do not occur naturally (52).

The most commonly used solid supports for antigen immobilization are 96-well plates, typically made of polystyrene, and beads, including magnetic or polymer beads (51). While 96-well plates were traditionally used for biopanning, beads have gained prominence in recent years due to their larger surface area for antigen binding, ease of recovery, and washing (51). Commercial kits are now available with beads pre-coated with streptavidin or neutravidin, facilitating the immobilization of biotinylated proteins. However, 76% of the publications still opted for the use of 96-well plates, likely due to their convenience, cost-effectiveness, and well-established protocols.

On the other hand, the biotin-streptavidin complex emerges as an alternative to this obstacle, offering the possibility of orienting the antigen as desired through the biotinylation process of the protein. However, the antigen’s characteristics, such as structure and purity, should be taken into account to determine the real need for this strategy.

After the first round of biopanning, phages displaying VHHs with target specificity are eluted and used to infect new bacterial cells. The newly infected cells produce phages displaying VHHs with higher specificity compared to the previous cycle. This amplification process leads to the enrichment of binding phages in each cycle (50). Several cycles of biopanning help reduce the variability of ligands and select for a specific subpopulation. However, a balance must be maintained in the number of cycles to avoid excluding viable ligands or selecting non-specific antibodies (50). Among the selected materials, 3 and 4 cycles of biopanning were the most used, aiming to obtain specific Nbs without excluding potential ligands.

At the end of the last biopanning cycle, the phages displaying VHHs are once again amplified in new bacterial cells. These phages are then precipitated, typically through centrifugation and the use of a polyethylene glycol solution with NaCl. Subsequently, the phages displaying VHHs are tested for their specificity and target binding potency using assays such as ELISA and MagPlex. VHHs with a positive and strong signal, usually three times greater than the control, are selected for sequencing. Sequenced VHHs are grouped into families based on the length of their CDR3 regions. Nbs within the same family originate from the same lineage of B lymphocytes, characterized by the same V-D-J rearrangement and somatic hypermutations that occur during affinity maturation (16, 17). Additionally, genetic manipulation processes, PCR, library construction, and isolation methods may also contribute to differences between Nb families (16).

As antigenic recognition is dependent on CDR3, Nbs from the same family will bind to the same antigen with potentially slight differences in affinities (16). Thus, the immune response generated by the antigen likely impacts the number of Nb families and the representation within each family. This variation may explain the differences observed in the number of families obtained across different materials.

Given the intricate interdependence among the variables in constructing the immune library and the final quality of the obtained nanobody, the Kd and IC_50_ values of nanobodies generated through specific genetic material amplification strategies, such as conventional PCR and Nested PCR, were scrutinized using statistical analysis to evaluate the presence of significant differences. The Mann-Whitney U test, with a predetermined significance level of 0.05, was employed for this purpose. The obtained results (p-value for K_d_ = 0.4668 and p-value for IC_50 = _0.0720) indicated the absence of statistically significant divergences in the K_d_ and IC_50_ values associated with different methodologies for the amplification of VHHs genes, compared to materials providing the K_d_ and IC_50_ values. Similarly, the K_d_ and IC_50_ values obtained from materials that underwent the biopanning process with direct adsorption of the antigen on the support were compared with those that involved biotinylation of the antigen, subjected to the same statistical analysis. The results revealed no statistically significant divergences for the IC_50_ values (p-value = 0.62017). However, concerning the analysis values, statistically significant differences were identified (p-value = 0.0018), leading to the rejection of the null hypothesis. This suggests that the manner in which the antigen is immobilized impacts the generation of antibodies, resulting in distinct affinities.

In this context, it was observed that the mean and median K_d_ values were significantly lower in materials that employed biotinylation of the antigen for conjugation with streptavidin and similar substances. These materials predominantly achieved affinities in the pM and sub-nM range. On the contrary, even though materials utilizing direct adsorption of the antigen on the support also attained affinities in the pM and sub-nM range, it was noted that these materials exhibited, on average and median, higher values compared to the first strategy, occasionally reaching relatively elevated K_d_ values. The dissociation constant serves as a key parameter reflecting the affinity of antibodies for the target. Therefore, a lower value of this constant corresponds to a greater affinity of the antibody. These results align with theoretical expectations, suggesting that the proficiency in targeting the antigen during the immobilization process indeed leads to enhanced recognition of relevant epitopes and superior binding efficiency. This approach appears to favor the generation of nanobodies with heightened affinity for the target.

### 5.4 Obtaining and testing nanobodies

The genes encoding the selected nanobodies proceed to the protein expression process. For this, the vector used in the phage display process is recovered and subcloned into an expression vector. Alternatively, the phagemid vector can be used for expression by transforming it into another non-suppressor bacterial strain. Both methods result in the production of recombinant Nbs with target specificity. Typically, these recombinant proteins have tailed that aid in the separation and purification process from the cell culture medium. The sequence encoding these tails is inherently present in most commercial vectors, occupying different positions on the plasmid. Generally, these sequences must be positioned at the end of the target sequence to prevent mutations or production of non-functional proteins. The polyhistidine tail (His-tag) is the most used among the tails present in expression vectors.

The purification of recombinant Nbs can be performed in various ways, but chromatographic processes are typically the most utilized, both on a small and large scale. The His-tag facilitates this purification process. Chromatographic columns containing metal ions that interact with histidine molecules are used. This method is known as immobilized metal affinity chromatography (IMAC). The recombinant Nbs containing the His-tag interact with the metallic resin of the column and are eluted at the end of the process. After protein purification, the subsequent steps depend on the intended application. For characterization tests and development of diagnostic tests, there is no requirement to remove the His-tag. However, for Nbs intended to produce biopharmaceuticals and *in vivo* neutralization tests, it is recommended to remove the tail. The presence of the His-tag in drugs can increase the risk of immunogenicity in patients, which can be detrimental to drugs (53). To remove the His-tag, enzymes that cleave the tail are added after the purification process. The choice of enzyme depends on the sequence present in the expression vector and the protein composition. Thrombins and enterokinases are commonly used (53).

Modifications can be made to the structure of Nbs with the aim of improving certain characteristics, such as half-life in circulation and bioavailability. These modifications can be made after the expression of monomeric nanobodies by adding flexible linkers. Alternatively, these modifications can be incorporated during the expression process, where the vector contains the sequence of the VHH monomers and the linker, resulting in the production of these recombinant constructs. This second method often requires different vectors and chromatographic purification processes.

After expression and purification of the Nbs, characterization tests are conducted. These assays determine molecular mass, crystalline structure, affinity for the target, interaction with other molecules (such as antigens), and physicochemical characteristics. Neutralization assays can also be performed to assess the ability of these Nbs to neutralize their targets. *In vitro* neutralization tests are initially carried out with cell cultures by infecting cells and subsequently adding Nbs. These cells are then used in other tests, such as flow cytometry and determination of viral load by PCR. After determining the neutralizing ability of the Nbs *in vitro*, *in vivo* tests can be conducted.

Other benefits arising from the reduced size of nanobodies include ease of genetic manipulation, cloning, and subsequent expression in a prokaryotic system, resulting in reduced production costs. However, large-scale production of recombinant proteins in this manner may introduce contaminants, such as endotoxins, into the final product. In addition to toxicity, these contaminants have negative impacts on the final immunogenicity of biotherapeutics and can also cause tissue damage. Therefore, the endotoxin limit for preclinical research in biotherapeutics is set at 5 Endotoxin Units (EU) per kilogram of body mass per hour (54). Although the removal of endotoxins presents a significant challenge for the biopharmaceutical industry, there are currently various methods for detecting and removing this contaminant, with affinity chromatography being the most widely used (54). It is relevant to note that both the production of nanobodies in prokaryotic systems and the production of monoclonal antibodies (mAbs) in cell culture can introduce contaminants into the medium during the biomanufacturing process. Awareness of these challenges is essential for the safe and effective advancement of antibody-based therapies, requiring rigorous quality control practices throughout the production process to ensure the safety and efficacy of biotherapeutics.

While biopanning and ELISA are valuable strategies for validating phage display libraries (25), there are some caveats and challenges associated with these techniques. Caveats for biopanning mostly include nonspecific binding, epitope masking, and limited diversity, while for ELISA, they encompass confirmation bias, false positives, and sensitivity issues. In general, while biopanning and ELISA are powerful tools for phage display validation, researchers must be aware of these caveats and take appropriate measures to optimize conditions, control for biases, and validate results using multiple approaches. Integrating these strategies into a comprehensive validation pipeline enhances the reliability of phage display nanobodies’ outputs.

Despite the numerous variables during biopanning, especially those related to antigen immobilization methods, it is crucial to consider that each choice, from the immunization process to molecular and phage display steps, can significantly influence the characteristics of expressed Nbs. However, promising results have been achieved, demonstrating the effectiveness of these approaches even in different experimental contexts (55–58). In studies employing the direct adsorption technique on microplates (55, 57), a significant difference in the affinity of the expressed Nbs was observed. For instance, one Nb achieved optimal K_d_ values, equal to 143 pM (57), while another exhibited a lower K_d_ value of 4.25 nM (55). Similarly, in studies opting for antigen biotinylation with immobilization on streptavidin-coated beads, a similar pattern was observed (56, 58). In this case, Nbs also reached optimal Kd values, ranging between 20 and 615 pM (58), while another set of Nbs achieved values of approximately 0.5 nM (56). Of the four materials analyzed, three did not detect cross-reactivity among the obtained Nbs. However, only one, conducted with the biotin-streptavidin complex, exhibited reactivity between SARS-CoV and SARS-CoV-2 viruses (56). Regarding SARS-CoV-2 neutralization, despite the different tests and constructs derived from different VHHs used in the materials, all obtained promising results.

In the face of infectious diseases, nanobodies (Nbs) have also demonstrated significant potential in recognizing and neutralizing several viruses. In addition to promising results obtained in the campaigns for selecting VHHs against SARS-CoV-2, other campaigns also deserve attention. Immobilizing CHIKV’s E1 and E2 proteins by direct adsorption on microplates resulted in obtaining VHHs with affinities in the pM range, without showing cross-reactivity with other Alphavirus family viruses, a significant challenge when generating specific antibodies against viruses within this family. In neutralization tests by these Nbs, the IC_50_ values ranged from 0.6 nM to 45.6 nM (59). However, immobilizing the VP1 protein of duck hepatitis A virus by direct adsorption on microplates led to the generation of VHHs that failed to neutralize the virus (60).

Another approach involves combining different antigen immobilization methods to ensure a comprehensive representation of the target epitopes during biopanning. For example, the NS1 protein of ZIKV was initially immobilized by direct adsorption on microplates in the first panning cycle, then the antigen was biotinylated and formed a complex with streptavidin in the second cycle, and finally, in the third cycle, NS1 was captured by llama IgG previously coated on the plate (61). The resulting VHHs were directed towards ZIKV detection assays, with only one of the clones showing cross-reactivity with other viruses in the same family (yellow fever, dengue, and West Nile virus), a considerable challenge within the flaviviridae family. Furthermore, the relative affinities of the Nbs were calculated by the nanobody concentrations causing 50% signal saturation (SC_50_), yielding promising results in the range of 1.5 to 8.2 ng/mL (61). These examples highlight the diversity of strategies in the VHH selection process by phage display, culminating in the generation of exceptional candidates for biopharmaceutical development.

## 6 Final considerations

After the discovery of nanobodies in the 1990s, they have been used in several areas of medicine and veterinary medicine. Their applications range from the treatment of diseases such as cancer, infectious and autoimmune diseases, to specific antigen detection tests *in vitro* (19). These tests have a wide range of capabilities, including the detection of infectious agents, cancer markers, contaminants such as pesticides and herbicides, and even allergens in food (16). Nanobodies also show great potential in *in vivo* diagnostic applications, acting as probes in imaging tests (17).

Starting from 2003, there has been a remarkable growth in the number of publications and patent applications involving VHHs (19). This trend continues to show significant growth, with approximately six clinical studies in progress in 2022 alone (62). The potential applications of nanobodies are further exemplified by the numerous studies conducted on SARS-CoV-2 as a target, representing 42% of the materials selected in the study. The virus responsible for the COVID-19 pandemic has spurred intensive scientific efforts, and one of the tools explored in the fight against the disease is the use of Nbs for treatment and diagnosis.

Some representative examples of the situations mentioned earlier include the Nbs currently in Phase I clinical trials conducted by Sanofi, SAR443765 and SAR444200. These pharmaceuticals consist of bispecific VHHs recognizing distinct targets, with the first intended for asthma treatment, recognizing TSLP and IL-13, while the second is directed at TCRαβ and GPC3 for the treatment of solid tumors (47). Ozoralizumab (Nanozora^®^), in Phase II/III by Taisho Pharmaceutical Co. Ltd, is a trivalent Nb recognizing TNFα, being tested for rheumatoid arthritis treatment (48). Envafolimab is an example of a humanized nanobody currently in Phase I, recognizing PD-L1 in solid tumors (49).

The reduced size of Nanobodies (Nbs) presents notable advantages, such as enhanced tissue penetration, the ability to cross the blood-brain barrier, and access to challenging epitopes. However, this characteristic also results in a reduced circulating half-life, associated with high clearance rates. To overcome these challenges, several strategies have been developed to make Nb-based therapy more viable. A key approach is the conjugation of VHHs into bivalent and trivalent forms. Additionally, some authors have explored the conjugation of selected VHHs with a specific VHH against serum albumin, providing a significant increase in the half-life of these constructs (63). The humanization of selected VHHs has also become a common practice to prevent potential immunogenicity scenarios, although the literature emphasizes the low immunogenicity caused by Nbs due to their reduced size. Humanization is often achieved by conjugating the selected VHH with the Fc portion of human IgG1. Concrete examples of these strategies are evident in Nbs currently in Phase clinical trials conducted (64–66). These initiatives illustrate the strategies adopted to overcome limitations associated with the size and half-life of Nanobodies in therapeutic applications. These modifications allow for further optimization of nanobody performance, combining key characteristics for their application in the healthcare field.

With the development and widespread use of phage display and biopanning techniques, the process of selecting target-specific nanobodies has become more streamlined. This has made it feasible to construct immune libraries of phages displaying specific nanobodies, thereby optimizing subsequent steps such as expression and purification. Similarly, to the expression of monomeric nanobodies, the production of VHH conjugates is easily achieved during expression, typically in prokaryotic systems. The advantages of producing nanobodies in prokaryotic cells include lower costs, ease of manipulation, high productivity, and scalability. Chromatographic processes are then employed to purify the recombinant nanobodies, which are also compatible with large-scale production, facilitating commercialization. It is important to note that ongoing research and advancements in nanobody engineering aim to address some of these limitations. As the field progresses, researchers continue to explore ways to optimize nanobodies for diverse applications and overcome their inherent challenges.

In conclusion, target-specific nanobodies can be efficiently obtained through camelid hyperimmunization and the construction of immune libraries. Moreover, with advancements in biotechnology, their recombinant production has made their application in the biopharmaceutical and diagnostic markets feasible. Given their significant potential for application in these sectors, as well as their ease of expression and modification through cloning processes, it can be concluded that nanobodies are a tool that contributes and will continue to contribute to the advancement of medicine.

## Author contributions

VM: Investigation, Methodology, Writing – original draft, Writing – review & editing. CP: Formal analysis, Methodology, Writing – original draft, Writing – review & editing. BB: Funding acquisition, Supervision, Writing – review & editing. RF: Conceptualization, Funding acquisition, Writing – original draft, Writing – review & editing.
