# Supplementary material for: Nanobodies: a promising approach to treatment of viral diseases
Source: Front Immunol. 2024 Jan 23;14:1303353. doi: 10.3389/fimmu.2023.1303353 (PMC10844482; doi:10.3389/fimmu.2023.1303353)
Supplement: Supplementary file 1 [file Table_1.docx]

Supplementary Material

Article Title

Nanobodies: A Promising Approach for the Treatment of Viral Diseases

Vitória Meneghetti Minatel^1^, Carlos Roberto Prudencio^2^, Rui Seabra Ferreira Junior^1,*^

^1^Center for the Study of Venoms and Venomous Animals (CEVAP), São Paulo State University (UNESP—Univ Estadual Paulista), Botucatu 18610-307, São Paulo, Brazil

^2^Immunology Center, Adolfo Lutz Institute, São Paulo, São Paulo State, Brazil

^3^Graduate Program in Tropical Diseases, Botucatu Medical School (FMB), São Paulo State University (UNESP—Univ Estadual Paulista), Botucatu 18618-687, São Paulo, Brazil;

*** Correspondence:**Corresponding Author
[rui.seabra@unesp.br](mailto:rui.seabra@unesp.br)
rui.seabra@unesp.br

Supplementary Table 1: List of indexed descriptors obtained from the DeCS platform.

| DeCS | | |
| --- | --- | --- |
| Descriptor in Portuguese | English descriptor | Alternative terms |
| Anticorpos de Domínio Único | Single-Domain Antibodies | VHH antibody; Single Domain Antibody; VHH antibodies; VHH fragments; VHH Immunoglobulin Fragments; VHH Immunoglobulin Fragments; VNAR Immunoglobulin Fragments; VHH fragments; |
| Bioprospecção | Bioprospecting | Biopanorâmica; |
| Biblioteca Gênica | Gene Library | DNA library; Gene Library; cDNA library; Genotheque; |
| Camelídeos | Camelids, New World | Alpaca; Alpacas; New World Camelids; Guanaco; Guanacos; Glama mud; Lama glama guanicoe; guanicoa mud; mud huts; Llama; Llamas; Vicuña paços; vicuna vicuna; Vicuna; Vicunas; |
| Técnicas de Visualização da Superfície Celular | Cell Surface Display Techniques | Cell Surface Display Technology; Display Technique, Phage; Display Techniques, Phage; Phage Display Technique; Phage Display Techniques; Technique, Phage Display; Techniques, Phage Display |

Supplementary Table 2: List of indexed descriptors on the MeSH platform.

| MeSH | |
| --- | --- |
| English descriptor | Alternative terms |
| Single-Domain Antibodies | Single-Domain Antibody; Antibody, Single-Domain; Single Domain Antibody; Immunoglobulin VL Domain Fragments; VL Domain Fragments; Immunoglobulin VH; Domain Fragments; VH Domain Fragments; VHH Immunoglobulin Fragments; VHH Antibodies; Nanobodies; VHH Antibody; Antibody, VHH; VHH Fragments; VNAR; Immunoglobulin Fragments; VNAR Fragments; |
| Bioprospecting | Biopanning; |
| Gene Library | Gene Libraries; Libraries, Gene; Library, Gene; DNA Library; DNA Libraries; Libraries, DNA; Library, DNA; cDNA Library; Libraries, cDNA; Library, cDNA; cDNA Libraries; |
| Camelids, New World | Camelid, New World; New World Camelid; New World Camelids; Lama glama; Llamas; Llama; Vicugna pacos; Alpacas; Alpaca; Lama pacos;Vicugna vicugna; Vicunas; Vicuna; Lama glama guanicoe; Guanacos; Guanaco; Lama guanicoe; |
| Cell Surface Display Techniques | Cell Surface Display Technology; Phage Display Techniques; Display Technique, Phage; Display Techniques, Phage; Phage Display Technique; Technique, Phage, Display Techniques, Phage Display; |

**Supplementary Table 3 – List of biopanning cycles used in the selected articles.**

| Biopanning cycles | Number of articles |
| --- | --- |
| 1 | 4 |
| 2 | 17 |
| 3 | 20 |
| 4 | 3 |

**Supplementary Table 4 - Data compiled on hyperimmunization of camelids, based on selected articles. Viruses have been grouped into families for comparison purposes.**

| Family | Vírus | Antigenic Form | Adjuvant | Route of administration | Animal | Immunizations | Interval between immunizations | Amount of Antigen | Reference |
| --- | --- | --- | --- | --- | --- | --- | --- | --- | --- |
| Coronaviridae | SARS-CoV-1 e MERS-CoV | Glycoprotein S - Structural | Gerbu LQ#3000 | Subcutaneous | Lhama | 6x | 7 days | 150 μg | [1] |
|  | SARS-CoV-2 | Glycoprotein S - Structural | Aluminum Hydroxide (Sigma) | Intradermally and subcutaneously in the pre-scapular region | Alpaca | 4x | 15 days | 400 μg | [2] |
|  | SARS-CoV-2 | Glycoprotein S - Structural | Freund's Complete and Incomplete Adjuvant | Uninformed | Alpaca | 3x | 7 days | 250 μg | [3] |
|  | SARS-CoV-2 | Glycoprotein S - Structural | Gerbu FAMA | Subcutaneous | Lhama | 6x | 7 days | 50 to 200 mg in different immunizations | [4] |
|  | SARS-CoV-2 | Glycoprotein S - Structural | Uninformed | Subcutaneous | Alpaca | 5x | 7 days | 33μg (protein S) and 100μg (RBD) | [5] |
|  | SARS-CoV-2 | Protein N - Structural | * | * | Lhama | 5x | 7 days | 100 μg | [6] |
|  | SARS-CoV-2 | Glycoprotein S - Structural | Freund's Complete and Incomplete Adjuvant | Subcutaneous | Alpaca | 4x | 15 days | 200 µg | [7] |
|  | SARS-CoV-2 | Glycoprotein S - Structural | Freund's Complete and Incomplete Adjuvant | Subcutaneous | Lhama | 5x | 7 days | 100 µg | [8] |
|  | SARS-CoV-2 | Glycoprotein S - Structural | Freund's Complete and Incomplete Adjuvant | Intramuscular and Subcutaneous | Alpaca | 3x | 15 days | 500 µg | [9] |
|  | Avian Coronavirus | Structural protein | Freund's Complete and Incomplete Adjuvant | Uninformed | bactrian camel | 5x | Uninformed | 2 mg | [10] |
|  | SARS-CoV-2 | Glycoprotein S - Structural | Gerbu | Subcutaneous | Alpaca | 4x | 4 days | 1 mg | [11] |
|  | SARS-CoV-2 | Glycoprotein S - Structural | GERBU Fama | Subcutaneously in the region of the scapula | Alpaca | 4x | 15 days | 200 µg | [12] |
|  | SARS-CoV-2 | Glycoprotein S - Structural | Gerbu | Subcutaneous | Alpaca | 3x | 4 days | 1 mg | [13] |
|  | SARS-CoV-2 | Glycoprotein S - Structural | Freund's Complete and Incomplete Adjuvant | Subcutaneous | Lhama | 6x | 15 days | 1 mg | [14] |
|  | SARS-CoV-2 | Glycoprotein S - Structural | Freund's Complete and Incomplete Adjuvant | Uninformed | bactrian camel | 5x | 5 days | uninformed | [15] |
|  | SARS-CoV-2 | Glycoprotein S - Structural | Aluminum Hydroxide (Sigma) | Intradermal and Subcutaneous | Alpaca | 3x | 45 days | 1 mg | [16] |
|  | SARS-CoV-2 | Interaction Protein | Gerbu P | Subcutaneous | Lhama | 6x | 7 days | 100 µg | [17] |
|  | SARS-CoV-2 | Structural protein | Freund's Complete and Incomplete Adjuvant | Uninformed | Alpaca | 3x | 16 days (*boost* 1) and 9 days (*boost* 2) | 150 μg | [18] |
|  | SARS-CoV-2 | Glycoprotein S - Structural | Freund's Complete Adjuvant | Subcutaneous | Alpaca | 9x | 15 days | Uninformed | [19] |
|  | SARS-CoV-2 | Glycoprotein S - Structural | Gerbu LQ#3000 | Intramuscular | Lhama | 2x | 28 days | 400 μg | [20] |
|  | Porcine Epidemic Diarrhea Virus (PEDV) | Structural protein | Freund's Complete and Incomplete Adjuvant | Uninformed | bactrian camel | 5x | 15 days | 5 mg | [21] |
| Flaviviridae | Dengue (DENV) | Proteína NS1 - Replicação | * | * | Lhama | 4x | uninformed | 50 μg | [22] |
|  | West Nile Virus (WNV) | Glycoprotein - Structural | Freund's Complete and Incomplete Adjuvant | Intramuscular | Lhama | 6x | 7 days | 200 µg (immunization 1) and 100 µg (immunization 2, 3, 4 and 5) | [23] |
|  | Zika Virus (ZIKV) | Glycoprotein - Replication | Freund's Incomplete Adjuvant | Subcutaneous | Lhama | 4x | 45 days | 150 µg | [24] |
|  | Bovine Viral Diarrhea Virus (BVDV) | Replication Protein | Freund's Complete Adjuvant | Subcutaneous | bactrian camel | 6x | 14 days | 5 mg | [25] |
| Togaviridae | Venezuelan Equine Encephalitis (VEEV) | Attenuated virus vaccine | - | * | Lhama | 4x | 14 days | - | [26] |
|  | Chikungunya (CHIKV) | Nucleoprotein NP - Structural | Uninformed | Uninformed | Lhama | 4x | 21 days | 100 μg | [27] |
|  | Western Equine Encephalitis (WEEV) | Inactivated virus vaccine | - | * | Lhama | * | * | - | [28] |
| Filoviridae | Ebola (EBV) | Glycoprotein - Structural | Incomplete Freund's adjuvant on last immunization only | Intramuscular | Alpaca | 4x | 45 days | 200 μg | [29] |
|  | Ebola (EBV) | Structural protein | * | * | Lhama | 4x | 21 days | 50 μg | [30] |
| Retroviridae | Human Immunodeficiency (HIV) | Human receptor protein | Uninformed | Uninformed | Lhama | 6x | Uninformed | Uninformed | [31] |
| Papillomaviridae | Human Papillomavirus 16 (HPV16) | Interaction protein | Freund's Complete and Incomplete Adjuvant | Subcutaneous | bactrian camel | 7x | 7 days | 1 mg | [32] |
|  | Human Papillomavirus 16 (HPV16) | Interaction protein | Freund's Complete and Incomplete Adjuvant | Uninformed | Lhama | 4x | 7 days | 100 μg (animal 1) and 250 μg (animal 2) | [33] |
| Arenaviridae | Lassa (LV) | Nucleoprotein NP - Structural | * | * | Lhama | 5x | 14 days | 100 μg | [34] |
| Paramyxoviridae | Peste des Petits Ruminants Virus (PPRV) | Attenuated vírus vaccine | - | Subcutaneous | Alpaca | 3x | 15 days | - | [35] |
| Bunyaviridae | Rift Valley fever vírus (RVFV) e Schmallenberg vírus (SBV) | Attenuated vírus vaccine | - | Intramuscular | Lhama | 3x | 60 days | 10^7^ TCID_50_ e 10^6^ TCID_50_ | [36] |
| Pneumoviridae | Human Respiratory Syncytial Virus (HRSV) | Structural protein | Poly (I:C) (Invivogen) and Gerbu LQ#3000 | Subcutaneous | Lhama | 6x | 7 days | 167 µg | [37] |
| Picornaviridae | Duck Hepatitis A (DHAV) | Attenuated vírus vaccine | - | Subcutaneous | bactrian camel | 6x | 15 days | - | [38] |
| Adenoviridae | Adenovirus | Attenuated vírus vaccine | - | Uninformed | bactrian camel | 4x | 15 days | 2 × 10^11^ PV/ml e 1 × 10^9^ /ml | [39] |
| Orthomyxoviridae | Influenza B (IVB) | Structural protein | Freund's Complete and Incomplete Adjuvant | Intramuscular in front and hind legs | Alpaca | 4x | 21 days | 50 µg | [40] |
| Nodaviridae | Nervous Necrosis Virus (NNV) | Attenuated vírus vaccine | Freund's Complete and Incomplete Adjuvant | Subcutaneous | Lhama | 4x | 15 days | 400 μg | [41] |
| Reoviridae | Mal de Río Cuarto vírus (MRCV) | Replication Protein | Freund's Complete and Incomplete Adjuvant | Uninformed | Lhama | 4x | 14 days | 100 µg | [42] |
| Hepeviridae | Swine Hepatitis E Virus (SHEV) | Replication Protein | Freund's Complete and Incomplete Adjuvant | Subcutaneous | bactrian camel | 5x | 15 days | 2mg | [43] |

* Immunization process carried out by Triple J Farms. TCID50 - Median Tissue Culture Infectious Dose. PV – Viral Particle.

| **Supplementary Table 5 – Data compiled from the selected articles, for the construction of the immune library of phages displaying VHHs. The essential steps in the process of building the library through hyperimmunization of camelids are summarized.** | | | | | | | | | |
| --- | --- | --- | --- | --- | --- | --- | --- | --- | --- |
| Family | Virus | RNA Extraction | cDNA Synthesis | PCR | Cloning | Phage display Vector | Helper Phage | Cell | Reference |
| Coronaviridae | SARS-CoV-1 and MERS-CoV | Total RNA from peripheral blood lymphocytes | Total RNA with oligo d(T) primers and reverse transcriptase | Convencional PCR | Restriction Enzyme Cloning with PstI and NotI | pMECS | VCSM13 | *E.coli* TG1 | [1] |
|  | SARS-CoV-2 | Total RNA from peripheral blood mononuclear cells | Total RNA with oligo d(T) and random primers and specific gene for alpaca IgG and reverse transcriptase | Convencional PCR | Restriction Enzyme Cloning with shRI | HQ2-2 | VCSM13 | *E.coli* TG1 | [2] |
|  | SARS-CoV-2 | Total RNA from peripheral blood mononuclear cells | Total RNA with oligo d(T) and random primers and reverse transcriptase | Convencional PCR | uninformed | phV1 | M13KO7 | *E.coli* TG1 | [3] |
|  | SARS-CoV-2 | Total RNA from peripheral blood lymphocytes | Total RNA with random primers and reverse transcriptase | Nested PCR | Restriction Enzyme Cloning | pMES4 | M13KO7 | *E.coli* TG1 | [4] |
|  | SARS-CoV-2 | Total RNA from peripheral blood lymphocytes | Total RNA with gene primers specific to the IgG CH2 domain | Nested PCR | Restriction Enzyme Cloning with BamHI and NotI | pHEN4 | M13KO7 | *E.coli* TG1 | [5] |
|  | SARS-CoV-2 | Total RNA from peripheral blood mononuclear cells | Total RNA with oligo d(T) primers and reverse transcriptase | Convencional PCR | Restriction Enzyme Cloning with SfiI | pECAN21 | Uninformed | *E. coli* XL1 Blue | [6] |
|  | SARS-CoV-2 | Total RNA from peripheral blood mononuclear cells | RT - PCR | Nested PCR | Restriction Enzyme Cloning with SfiI | pCANTAB5E | Uninformed | *E.coli* TG1 | [7] |
|  | SARS-CoV-2 | Total RNA from peripheral blood mononuclear cells | Total RNA with random primers and reverse transcriptase | Nested PCR | Restriction Enzyme Cloning with NcoI and NotI | pHEN2 | M13KO7 | *E.coli* TG1 | [8] |
|  | SARS-CoV-2 | Total RNA from peripheral blood mononuclear cells | Total RNA with oligo d(T) primers and reverse transcriptase | Convencional PCR | Gibson Assembly | pR2 | KM13 | *E.coli* TG1 | [9] |
|  | Avian Coronavirus | Total RNA from peripheral blood mononuclear cells | Reverse transcription | Nested PCR | Restriction Enzyme Cloning with PstI and NotI | pMECS | M13KO7 | *E.coli* TG1 | [10] |
|  | SARS-CoV-2 | Peripheral blood lymphocyte mRNA | Reverse transcription | Nested PCR | Restriction Enzyme Cloning with BspQI | pDXinit | M13KO7 | *E. coli* SS320 | [11] |
|  | SARS-CoV-2 | Total RNA from peripheral blood lymphocytes | Total RNA with oligo d(T) and random primers and specific gene for alpaca IgG and reverse transcriptase | Convencional PCR | Gibson Assembly | pHEN | VCSM13 | *E.coli* TG1 | [12] |
|  | SARS-CoV-2 | RNAm from peripheral blood lymphocytes | Reverse transcription with commercial kit | Nested PCR | Restriction Enzyme Cloning with BspQI | pDXinit | M13KO7 | *E. coli* SS320 | [13] |
|  | SARS-CoV-2 | Total RNA from peripheral blood mononuclear cells | Total RNA with random primers and reverse transcriptase | Nested PCR | Restriction Enzyme Cloning with PstI-HF and BstEII-HF | pMES4 | VCSM13 | *E.coli* TG1 | [14] |
|  | SARS-CoV-2 | Total RNA from peripheral blood lymphocytes | Reverse transcription with commercial kit | Nested PCR | Restriction Enzyme Cloning with PstI and NotI | pMECS | Uninformed | *E.coli* TG1 | [15] |
|  | SARS-CoV-2 | Total RNA from peripheral blood mononuclear cells | Total RNA with oligo d(T) primers and random primers and reverse transcriptase | Convencional PCR | Restriction Enzyme Cloning with Not1 and Asc1 | pCANTAB 5E | M13 | *E.coli* TG1 | [16] |
|  | SARS-CoV-2 | Total RNA from peripheral blood lymphocytes | Total RNA with oligo d(T) primers and reverse transcriptase | Convencional PCR | Restriction Enzyme Cloning with PstI and NotI | pMECS | Uninformed | *E.coli* TG1 | [17] |
|  | SARS-CoV-2 | Total RNA from peripheral blood lymphocytes | Reverse transcription | Nested PCR | Restriction Enzyme Cloning with SfiI and NotI | pHEN6 | VCSM13 | *E.coli* TG1 | [18] |
|  | SARS-CoV-2 | Total RNA from peripheral blood mononuclear cells | Total RNA with random primers and reverse transcriptase | Nested PCR | Restriction Enzyme Cloning with PstI- and BstEII- | pMES4 | Uninformed | *E.coli* TG1 | [19] |
|  | SARS-CoV-2 | Total RNA from peripheral blood mononuclear cells | Total RNA with specific primers and reverse transcriptase | Nested PCR | Restriction Enzyme Cloning with SfiI | pADL-23c | M13KO7 | *E.coli* TG1 | [20] |
|  | Porcine Epidemic Diarrhea Virus (PEDV) | Total RNA from peripheral blood lymphocytes | Total RNA with oligo d(T) primers and random primers and reverse transcriptase | Nested PCR | Restriction Enzyme Cloning with PstI and NotI | pCANTAB 5E | M13KO7 | *E.coli* TG1 | [21] |
| Flaviviridae | Dengue (DENV) | Total RNA from peripheral blood lymphocytes | RT - PCR | Convencional PCR | Restriction Enzyme Cloning with SfiI | pECAN21 | M13KO7 | *E. coli* XL1 Blue | [22] |
|  | West Nile Virus (WNV) | Total RNA from peripheral blood mononuclear cells | Total RNA with specific primers and reverse transcriptase | Convencional PCR | Restriction Enzyme Cloning with SfiI | pJB12 | M13KO71pIII | *E. coli* XL1 Blue | [23] |
|  | Zika Virus (ZIKV) | Total RNA from peripheral blood mononuclear cells | Total RNA with random primers and reverse transcriptase | Convencional PCR | Restriction Enzyme Cloning with SfiI | pComb3X | M13KO7 | *E. coli* ER2738 | [24] |
|  | Bovine Viral Diarrhea Virus (BVDV) | Total RNA from peripheral blood mononuclear cells | RT - PCR | Nested PCR | Uninformed | pCANTAB 5E | Uninformed | Não informado | [25] |
| Togaviridae | Venezuelan Equine Encephalitis (VEEV) | Total RNA from peripheral blood lymphocytes | Total RNA with oligo d(T) primers and reverse transcriptase | Convencional PCR | Restriction Enzyme Cloning with SfiI | pECAN21 | M13KO7 | *E. coli* XL1 Blue | [26] |
|  | Chikungunya (CHIKV) | Total RNA from peripheral blood lymphocytes | Total RNA with oligo d(T) primers and reverse transcriptase | Convencional PCR | Restriction Enzyme Cloning with SfiI | pECAN21 | Uninformed | *E. coli* XL1 Blue | [27] |
|  | Western Equine Encephalitis (WEEV) | Total RNA from buffy coat cells | Total RNA with oligo d(T) primers and reverse transcriptase | Convencional PCR | Restriction Enzyme Cloning with SfiI | pECAN21 | Uninformed | *E. coli* XL1 Blue | [28] |
| Filoviridae | Ebola (EBV) | B lymphocyte mRNA | RT - PCR | Nested PCR | Restriction Enzyme Cloning with SfiI | pHEN1 | M13KO7 | *E.coli* TG1 | [29] |
|  | Ebola (EBV) | Total RNA from peripheral blood mononuclear cells | Total RNA with oligo d(T) primers and reverse transcriptase | Nested PCR | Restriction Enzyme Cloning with SfiI | pECAN126 | Uninformed | *E. coli* HBV88 | [30] |
| Retroviridae | Human Immunodeficiency  (HIV) | Total RNA from peripheral blood lymphocytes | RT - PCR | Convencional PCR | Restriction Enzyme Cloning with | uninformed | Uninformed | *E.coli* TG1 | [31] |
| Papillomaviridae | Human Papillomavirus 16 (HPV16) | Total RNA from peripheral blood lymphocytes | Total RNA with oligo d(T) primers and reverse transcriptase | Nested PCR | Restriction Enzyme Cloning with EcoRI and HindIII | Phages T7 Select | - | *E.coli* BLT5403 | [32] |
|  | Human Papillomavirus 16 (HPV16) | Total RNA from peripheral blood lymphocytes | Total RNA with oligo d(T) primers and random primers and reverse transcriptase | Nested PCR | Restriction Enzyme Cloning with SfiI | pMED1 | M13KO7 | *E.coli* TG1 | [33] |
| Arenaviridae | Lassa (LV) | Total RNA from buffy coat cells | Total RNA with oligo d(T) primers and reverse transcriptase | Convencional PCR | Restriction Enzyme Cloning with SfiI | pECAN21 | Uninformed | *E. coli* XL1 Blue | [34] |
| Paramyxoviridae | Peste des Petits Ruminants Virus (PPRV) | Total RNA from peripheral blood lymphocytes | Total RNA with oligo d(T) primers and reverse transcriptase | Nested PCR | Restriction Enzyme Cloning with SapI | pMECS | M13KO7 | *E.coli* TG1 | [35] |
| Bunyaviridae | Rift Valley fever vírus (RVFV) and Schmallenberg vírus (SBV) | Total RNA from peripheral blood lymphocytes | Reverse transcription of mRNA with commercial kit | Convencional PCR | Restriction Enzyme Cloning with PstI and NotI | pRL144 | Uninformed | *E. coli* JM109 | [36] |
| Pneumoviridae | Human Respiratory Syncytial Virus (HRSV) | Total RNA from peripheral blood lymphocytes | Total RNA with oligo d(T) primers and reverse transcriptase | Nested PCR | Restriction Enzyme Cloning with PstI and NotI | pHEN4 | VCSM13 | *E.coli* TG1 | [37] |
| Picornaviridae | Duck Hepatitis A (DHAV) | Total RNA from peripheral blood mononuclear cells | Total RNA with oligo d(T) primers and reverse transcriptase | Nested PCR | Uninformed | pCANTAB 5E | Uninformed | *E.coli* TG1 | [38] |
| Adenoviridae | Adenovirus | Total RNA from peripheral blood mononuclear cells | Total RNA with random primers and reverse transcriptase | Nested PCR | Restriction Enzyme Cloning with NcoI and NotI | pMECS | M13KO7 | *E.coli* TG1 | [39] |
| Orthomyxoviridae | Influenza B (IVB) | Total RNA from peripheral blood mononuclear cells | Total RNA with oligo d(T) primers and reverse transcriptase | Nested PCR | Restriction Enzyme Cloning with NcoI and NotI | pNIBS-1 | Uninformed | *E.coli* TG1 | [40] |
| Nodaviridae | Nervous Necrosis Virus (NNV) | Total RNA from peripheral blood lymphocytes | Total RNA with oligo d(T) primers and reverse transcriptase | Nested PCR | Restriction Enzyme Cloning with Sfi I and Not I | pHEN1 | M13KO7 | *E.coli* TG1 | [41] |
| Reoviridae | Mal de Río Cuarto vírus (MRCV) | Total RNA from peripheral blood mononuclear cells | Total RNA with oligo d(T) primers and reverse transcriptase | Nested PCR | Restriction Enzyme Cloning with PstI and NotI | pMECS | M13KO7 | *E.coli* TG1 | [42] |
| Hepeviridae | Swine Hepatitis E Virus (SHEV) | Total RNA from peripheral blood lymphocytes | Reverse transcription | Nested PCR | Restriction Enzyme Cloning with PstI and NotI | pMECS | M13KO7 | *E.coli* TG1 | [43] |

| **Supplementary Table 6 – Summary of the Nbs ligand selection process by the phage display and biopanning steps. The supports used in biopanning are described, the form of immunization of the antigens on this support, the amount of these proteins used, the number of cycles performed, the method of detecting the ligands and the number of families obtained in each study.** | | | | | | | | |
| --- | --- | --- | --- | --- | --- | --- | --- | --- |
| Family | Virus | *Biopanning* | Antigen adsortion | Amount of antigen | Rounds | Binding validation | Diversity | Reference |
| Coronaviridae | SARS-CoV-1 e MERS-CoV | 96-well plate | Direct adsorption | 20 μg | 2 | ELISA with periplasmic extract (soluble Nb) | 7 (MERS-CoV) and 5 (SARS-CoV-1) | [1] |
|  | SARS-CoV-2 | Magnetic beads | Direct adsorption | Uninformed | 2 | ELISA with bacterial supernatant | Uninformed | [2] |
|  | SARS-CoV-2 | 96-well plate | Direct adsorption | 50 μg/mL | 3 | ELISA with bacterial supernatant | Uninformed | [3] |
|  | SARS-CoV-2 | 96-well plate | Neutravidin – Biotin | 10 μg/mL | 2 | ELISA with periplasmic extract (soluble Nb) | 28 (alpaca 1) and 76 (alpaca 2) | [4] |
|  | SARS-CoV-2 | Streptavidin beads | Streptavidin - Biotin | Decreasing concentrations during biopanning | 4 | Immunofluorescence on Transfected HeLa Cells | 59 | [5] |
|  | SARS-CoV-2 | 96-well plate | Direct adsorption | Uninformed | 2 | MagPlex | 5 | [6] |
|  | SARS-CoV-2 | 96-well plate | Direct adsorption | Uninformed | 4 | ELISA with bacterial supernatant | - | [7] |
|  | SARS-CoV-2 | Streptavidin beads | Streptavidin - Biotin | 1 µg | 1 | Uninformed | 13 | [8] |
|  | SARS-CoV-2 | 96-well plate | Direct adsorption | 100 µg/mL (cycle 1) and 20 µg/mL (cycle 2) | 2 | ELISA with bacterial supernatant | 7 | [9] |
|  | Avian Coronavirus | 96-well plate | Direct adsorption | 30 µg (cycle 1); 10 µg (cycle 2) and 5 µg (cycle 3) | 3 | ELISA with bacterial supernatant | 7 | [10] |
|  | SARS-CoV-2 | 96-well plate (ciclo 1) and Streptavidin beads (ciclo 2 and 3) | Neutravidin - Biotin | 50 nM | 3 | ELISA with periplasmic extract (soluble Nb) | 28 | [11] |
|  | SARS-CoV-2 | Streptavidin beads (ciclo 1) magnetic beads (ciclo 2) | Streptavidin - Biotin (ciclo 1) and Direct adsorption (ciclo 2) | Uninformed | 2 | ELISA with periplasmic extract (soluble Nb) | - | [12] |
|  | SARS-CoV-2 | 96-well plate (ciclo 1) and Streptavidin beads (ciclo 2 and 3) | Neutravidin – Biotin (ciclo 1) and Streptavidin - Biotin (ciclo 2 and 3) | 50 nM (cycle 1 and 2) and 5 nM (cycle 3) | 3 | ELISA with periplasmic extract (soluble Nb) | 28 | [13] |
|  | SARS-CoV-2 | 96-well plate | Direct adsorption | 100 μg/mL | 1 (RBD) and 2 (Spike) | ELISA with periplasmic extract (soluble Nb) | 192 | [14] |
|  | SARS-CoV-2 | 96-well plate | Direct adsorption | 100 µg/mL | 3 | ELISA with periplasmic extract (soluble Nb) | 4 | [15] |
|  | SARS-CoV-2 | 96-well plate | Direct adsorption | 10 μg/mL | 2 | ELISA with periplasmic extract (soluble Nb) | Uninformed | [16] |
|  | SARS-CoV-2 | 96-well plate | Direct adsorption | 100 µg/mL | 3 | ELISA with periplasmic extract (soluble Nb) | 18 | [17] |
|  | SARS-CoV-2 | 96-well plate | Direct adsorption | Uninformed | 3 | ELISA with bacterial supernatant | Uninformed | [18] |
|  | SARS-CoV-2 | Magnetic beads | Direct adsorption | Uninformed | 1 | Uninformed | 18 | [19] |
|  | SARS-CoV-2 | Streptavidin beads | Streptavidin - Biotin | 50 nM (cycle 1) and 2 nM (cycle 2) | 2 | ELISA by inhibition and ELISA by competition | Uninformed | [20] |
|  | Porcine Epidemic Diarrhea Virus (PEDV) | 96-well plate | Direct adsorption | 100 µg/mL | 3 | ELISA with periplasmic extract (soluble Nb) | 3 | [21] |
| Flaviviridae | Dengue (DENV) | 96-well plate | Direct adsorption | 5 μg/mL | 3 | MagPlex | 5 | [22] |
|  | West Nile Virus (WNV) | 96-well plate | Direct adsorption | 1μg | 1 | Western blot | 20 | [23] |
|  | Zika Virus (ZIKV) | 96-well plate | Direct adsorption (cycle 1); Streptavidin - Biotin (cycle 2) and immunized llama IgG (cycle 3) | 1 µg/mL | 3 | ELISA with periplasmic extract (soluble Nb) | 22 | [24] |
|  | Bovine Viral Diarrhea Virus (BVDV) | 96-well plate | Direct adsorption | Uninformed | 3 | ELISA with bacterial supernatant | 8 | [25] |
| Togaviridae | Venezuelan Equine Encephalitis (VEEV) | 96-well plate | Direct adsorption | 30 µg/mL | 3 | ELISA with bacterial supernatant | 5 | [26] |
|  | Chikungunya (CHIKV) | 96-well plate | Direct adsorption | 30 μg/mL (VLPs) and 5 μg/mL (rE1) | 2 | ELISA with bacterial supernatant | 4 | [27] |
|  | Western Equine Encephalitis (WEEV) | 96-well plate | Direct adsorption | Uninformed | 3 | ELISA e MagPlex com sobrenadante bacteriano | 5 | [28] |
| Filoviridae | Ebola (EBV) | 96-well plate | Direct adsorption | Uninformed | 2 | ELISA with bacterial supernatant | Uninformed | [29] |
|  | Ebola (EBV) | 96-well plate | Direct adsorption | 1μg/mL | 2 | ELISA with bacterial supernatant | Uninformed | [30] |
| Retroviridae | Human Immunodeficiency  (HIV) | Unformed | Unformed | 1 μg | 2 | Uninformed | 10 | [31] |
| Papillomaviridae | Human Papillomavirus 16 (HPV16) | Agarose beads | Direct adsorption | Uninformed | 3 | ELISA with bacterial supernatant | 4 | [32] |
|  | Human Papillomavirus 16 (HPV16) | 96-well plate | Indirect adsorption to maltose binding protein (MBP) | 5 μg | 2 | ELISA with bacterial supernatant | 26 | [33] |
| Arenaviridae | Lassa (LV) | 96-well plate | Direct adsorption | 10 µg/mL | 3 | ELISA and MagPlex with bacterial supernatant | 4 | [34] |
| Paramyxoviridae | Peste des Petits Ruminants Virus (PPRV) | 96-well plate | Direct adsorption | 2 µg | 4 | ELISA with periplasmic extract (soluble Nb) | 3 | [35] |
| Bunyaviridae | Rift Valley fever vírus (RVFV) e Schmallenberg vírus (SBV) | 96-well plate | Streptavidin - Biotin | Uninformed | 3 | ELISA with bacterial supernatant | 15 | [36] |
| Pneumoviridae | Human Respiratory Syncytial Virus (HRSV) | 96-well plate | Direct adsorption | Uninformed | 2 | ELISA with periplasmic extract (soluble Nb) | 12 | [37] |
| Picornaviridae | Duck Hepatitis A (DHAV) | 96-well plate | Direct adsorption | 100 µg/mL (cycle 1); 80 μg/ml (cycle 2) and 40 μg/ml (cycle 3) | 3 | ELISA with bacterial supernatant | 1 | [38] |
| Adenoviridae | Adenovirus | 96-well plate | Direct adsorption | 2 × 10^7^ viral particle | 2 | ELISA with bacterial supernatant | - | [39] |
| Orthomyxoviridae | Influenza B (IVB) | Imunotubes | Direct adsorption | 10 µg/mL | 2 | Uninformed | 13 | [40] |
| Nodaviridae | Nervous Necrosis Virus (NNV) | 96-well plate | Direct adsorption | Uninformed | 3 | ELISA with bacterial supernatant | 6 | [41] |
| Reoviridae | Mal de Río Cuarto vírus (MRCV) | 96-well plate | Direct adsorption | 100 µg/mL | 3 | ELISA with bacterial supernatant | 12 | [42] |
| Hepeviridae | Swine Hepatitis E Virus (SHEV) | 96-well plate | Direct adsorption | 1 µg | 3 | ELISA with periplasmic extract (soluble Nb) | 8 | [43] |

| **Supplementary Table 7 – Process for obtaining and testing previously selected Nbs binders. The expression vectors employed in this process, the transformed cells, the Nbs purification method, the description of the modifications carried out in the antibodies after their expression, the characterization assays and the neutralization tests are present.** | | | | | | | | |
| --- | --- | --- | --- | --- | --- | --- | --- | --- |
| Family | Vírus | Expression vector | Transformed Cell | Purification | Nbs modification | Validation assays | Neutralization assays | References |
| Coronaviridae | SARS-CoV-1 e MERS-CoV | pKai61 | *Pichia pastoris* GS115 | Affinity chromatography with HisTrap column | (VHH)_2_ | ELISA; Crystalline structure; Cryo-electron microscopy and BLI | in vitro neutralization | [1] |
|  | SARS-CoV-2 | pHEN | *E. coli* BL21 | Affinity chromatography with Ni-NTA column and size exclusion | VHH-(hIgG)Fc | Crystalline structure; Cryo-electron microscopy and BLI | In vitro neutralization in Vero E6 cells and flow cytometry | [2] |
|  | SARS-CoV-2 | pcDNA3.4 | Mammalian cells 293F | Affinity chromatography with Ni-NTA column and size exclusion | VHH- (hIgG)Fc; (VHH)_2;_ (VHH)_3_ and (VHH)_4_ | ELISA and BLI | In vitro neutralization in Vero E6 cells and viral quantification by RT-PCR | [3] |
|  | SARS-CoV-2 | pMES4 | *E. coli* WK6 | Affinity chromatography with HisTrap column | VHH_1_-(hIgG)Fc-VHH_2_ | ELISA; FRET; BLI; ELISA; Crystalline structure; Cryo-electron microscopy | In vitro neutralization; Flow cytometry and in vivo neutralization | [4] |
|  | SARS-CoV-2 | pHEN4 | *E. coli* Shuffle Express and *E. coli* NEB Express | Affinity chromatography with Ni-NTA column | VHH_1_-VHH_2_-VHH_3_ | Crystalline structure; Cryo-electron microscopy and BLI | In vitro neutralization in Vero E6 cells and viral quantification by RT-PCR | [5] |
|  | SARS-CoV-2 | pET22b | *E. coli* Turner (DE3) | Affinity chromatography with immobilized metal ions | (VHH)_2_ and VHH_1_-VHH_2_ | Circular dichroism; SPR and MagPlex | - | [6] |
|  | SARS-CoV-2 | pCold I | *E. coli* | Affinity chromatography with Ni-NTA column | - | ELISA and BLI | In vitro neutralization and flow cytometry | [7] |
|  | SARS-CoV-2 | pHEN2 | *E. coli* BL21 | Affinity chromatography with HisTrap column | - | BLI; SPR; Melting temperature; Circular dichroism and ELISA | In vitro neutralizing Ab detection; In vitro neutralization and flow cytometry | [8] |
|  | SARS-CoV-2 | Uniformed | Mammalian cells 293F | Protein A | VHH- (hIgG)Fc | Circular dichroism; SPR and ELISA | In vitro neutralization in Vero E6 cells | [9] |
|  | Avian Coronavirus | pCMV-N1-vHRP | Mammalian cells HEK293T | Uninformed | - | ELISA; *Western Blotting* and IFA | - | [10] |
|  | SARS-CoV-2 | pSbinit | *E. coli* MC1061 | Ni-NTA Beads | - | ELISA; BLI; Size Exclusion Chromatography and Crystallization | In vitro neutralization in Vero E6 cells | [11] |
|  | SARS-CoV-2 | pHEN6 | *E. coli* BL21 | Size Exclusion Chromatography and Affinity chromatography with Ni-NTA column | (VHH)_2_ and VHH_1_-VHH_BSA_ | SPR; Epitope Mapping and Competitive Assay | In vitro neutralization in HEK293 cells; Flow cytometry; In vivo neutralization and RT-PCR | [12] |
|  | SARS-CoV-2 | pSbinit | *E. coli* MC1061 | Affinity chromatography with Ni-NTA column | VHH- (hIgG)Fc | ELISA; Size exclusion chromatography; BLI; X-ray crystallization and diffraction | In vitro neutralization in HEK293 cells | [13] |
|  | SARS-CoV-2 | pMES4 | *E. coli* WK6 | *Capturem His-tagged* ® e *cOmplete His-tag purification®* | VHH- (hIgG)Fc and (VHH)_3_- (hIgG)Fc | ELISA; BLI; Competitive assay and cryo-electron microscopy | In vitro neutralization; microplate neutralization | [14] |
|  | SARS-CoV-2 | pMECS | *E. coli* WK6 | Affinity chromatography with Ni-NTA | - | ELISA | - | [15] |
|  | SARS-CoV-2 | pLVX-IRES- | Mammalian cells 293F | Affinity chromatography with column Ni-NTA | (VHH)_2_ - (hIgG)Fc and (VHH)_2_ | ELISA and BLI | In vitro neutralization and FFA | [16] |
|  | SARS-CoV-2 | pMECS | *E. coli* WK6 | Affinity chromatography with immobilized metal ions and molecular exclusion | - | ELISA; Crystal structure and Nuclear magnetic resonance spectroscopy; | - | [17] |
|  | SARS-CoV-2 | pASK | *E. coli* BL21 | Affinity chromatography with a Strep-Tactin column followed by Molecular Exclusion | - | ELISA; Mass spectrometry and SPR | In vitro neutralization in FRhK4 cells and histomorphological analyzes | [18] |
|  | SARS-CoV-2 | pMES4 | *E. coli* BL21 | Affinity chromatography with HisTrap column | (VHH)_2_ | ELISA; Western Blotting; BLI; Immunochromatography; Crystalline structure; Cryo-electron microscopy | In vitro neutralization in Vero E6 cells and flow cytometry | [19] |
|  | SARS-CoV-2 | pOPINO | *E. coli* WK6 and Mammalian cells Expi293 | Affinity chromatography with HisTrap column and molecular exclusion | VHH- (hIgG)Fc e (VHH)_3_ | SPR; ITC; Crystal structure and cryo-electron microscopy | In vitro neutralization in Vero E6 cells and in vivo neutralization | [20] |
| Flaviviridae | Porcine Epidemic Diarrhea Virus (PEDV) | pET21b | *E. coli* BL21 | Affinity chromatography with column Ni-NTA | - | ELISA | - | [21] |
|  | Dengue (DENV) | pET22b | *E. coli* Turner (DE3) | Affinity chromatography with column Ni-*Sepharose* | - | MagPlex; SPR and Circular Dichroism | - | [22] |
|  | West Nile Virus (WNV) | pQE30 | *E. coli* Shuffle | Affinity chromatography with *His-Mag sepharose Ni beads* | - | *Dot Blot*; *on cell* ELISA and *Western Blotting* | In vitro neutralization in HEK293 cells; Hemolytic activity and toxicity assays | [23] |
|  | Zika Virus (ZIKV) | pINQ | *E. coli* BL21 | Affinity chromatography with column Ni-NTA | - | ELISA | - | [24] |
|  | Bovine Viral Diarrhea Virus (BVDV) | pTRIP-CMV- | Mammalian cells MDBK | Affinity chromatography with Ni-NTA | - | *Western Blotting; RT-qPCR and Viral Titration* | - | [25] |
| Togaviridae | Venezuelan Equine Encephalitis (VEEV) | pET22b | *E. coli* Turner (DE3) | Affinity chromatography with immobilized metal ions | (VHH)_2_ and VHH_1_-VHH_2_ | Circular dichroism | in vitro neutralization | [26] |
|  | Chikungunya (CHIKV) | pET22b | *E. coli* Turner (DE3) | Affinity chromatography with immobilized metal ions | - | Circular dichroism; SPR; MagPlex; Western Blotting and ELISA | in vitro neutralization | [27] |
|  | Western Equine Encephalitis (WEEV) | pET22b | *E. coli* Turner (DE3) | Affinity chromatography with column Ni-*Sepharose* | - | SPR; Melting Temperature and MagPlex | - | [28] |
| Filoviridae | Ebola (EBV) | pHEN1 | *E. coli* BL21 | Affinity chromatography with column Co-Agarose | VHH- (hIgG)Fc | ELISA indirect | In vitro neutralization in Vero E6 cells and in vivo neutralization | [29] |
|  | Ebola (EBV) | pECAN126 | Mammalian cells Vero E6 | Affinity chromatography with immobilized metal ions and molecular exclusion | (VHH)_2_ | ELISA and *Western Blotting*; | - | [30] |
| Retroviridae | Human Immunodeficiency  (HIV) | Uninformed | Mammalian cells HEK293 | Affinity chromatography with HisTrap column | VHH- (hIgG)Fc | ELISA; Analysis of binding by competition in Jurkat cells; Calcium immobilization assay and epitope mapping | Internalization test; Cell migration assay; Analysis of cell morphology and analysis of viral replication; | [31] |
| Papillomaviridae | Human Papillomavirus 16 (HPV16) | pET28a (+) | *E. coli* BL21 | Affinity chromatography with Ni-*Sepharose* | - | ELISA and MTT | - | [32] |
|  | Human Papillomavirus 16 (HPV16) | pSJF2H | *E. coli* TG1 | Affinity chromatography with immobilized metal ions | - | ELISA; SPR and *Western Blotting* | - | [33] |
| Arenaviridae | Lassa (LV) | pET22b | *E. coli* Turner (DE3) | Affinity chromatography with immobilized metal ions | - | Circular dichroism; SPR e MagPlex | - | [34] |
| Paramyxoviridae | Peste des Petits Ruminants Virus (PPRV) | pMECS | *E. coli* WK6 | Uninformed | - | ELISA | - | [35] |
| Bunyaviridae | Rift Valley fever vírus (RVFV) e Schmallenberg vírus (SBV) | pRL188 | *S. cerevisiae* | Affinity chromatography column Ni-NTA | VHH- (hIgG)Fc and (VHH)_2_ | SDS-PAGE; ELISA and IPMA | In vitro neutralization in Vero E6 cells and in vivo neutralization | [36] |
| Pneumoviridae | Human Respiratory Syncytial Virus (HRSV) | pKai61 | *P. pastoris* GS115 | Uninformed | - | SPR and Crystal Structure | In vitro neutralization and flow cytometry | [37] |
| Picornaviridae | Duck Hepatitis A (DHAV) | pET28a | *E. coli* Rosetta (DE3) | Affinity chromatography with column Ni-NTA | - | ELISA; IFA and epitope mapping | in vitro neutralization | [38] |
| Adenoviridae | Adenovirus | pET-25b (+) | *E.coli* BL21 | Affinity chromatography with column Ni-NTA | - | - | - | [39] |
| Orthomyxoviridae | Influenza B (IVB) | pNIBS-1 | *E. coli* WK6 | Affinity chromatography with column Ni-NTA | - | SPR and Display in Yeast | In vitro neutralization in HEK293 cells | [40] |
| Nodaviridae | Nervous Necrosis Virus (NNV) | pET-25b (+) | *E. coli* Rosetta (DE3) | Affinity chromatography with column Ni-NTA | - | ELISA and *Western Blotting* | in vitro neutralization | [41] |
| Reoviridae | Mal de Río Cuarto vírus (MRCV) | pMECS | *E. coli* WK6 | Affinity chromatography with immobilized metal ions and molecular exclusion | (VHH)_2_ | ELISA; SPR; *Western Blotting*; *native* -PAGE and SDS-PAGE | - | [42] |
| Hepeviridae | Swine Hepatitis E Virus (SHEV) | pET21b | *E. coli* BL21 | Affinity chromatography with HisTrap column | - | SDS-PAGE; IFA; *Western Blotting* and ELISA | Flow cytometry; Quantification of viral load by RT-PCR; In vivo neutralization and electron microscopy of tissues | [43] |

VHH – Variable Heavy Domain of Heavy Chain Antibody; (VHH)2 – VHH homodimer; VHH- (hIgG)Fc – VHH conjugated with the Fc portion of human IgG; VHH1-VHH2 - VHH heterodimer; (VHH)3 – VHH homotrimers; (VHH)2 - (hIgG)Fc – VHH homodimer conjugated with the Fc portion of human IgG; (VHH)3- (hIgG)Fc – VHH homotrimer conjugated with the Fc portion of human IgG; VHH1-VHHBSA – Heterodimer of VHH with VHH specific to human serum albumin; VHH1-VHH2-VHH3 - VHH heterotrimer; (VHH)4 – VHH homotetramer; ELISA - Enzyme Linked Immunosorbent Assay; BLI - Biolayer Interferometry; FRET - Fluorescence Resonance Energy Transfer; SPR – Surface Plasmon Resonance; IFA – Immunofluorescence Focus Test; IPMA - Immunoperoxidase monolayer assay; ITC - Isothermal titration calorimetry; FFA - Focus forming assay; MTT - (3-[4,5-dimethylthiazol-2-yl]-2,5-diphenyl tetrazolium bromide); SDS-PAGE - Sodium Dodecyl Sulfate-PolyAcrylamide Gel Electrophoresis; RT-PCR – Reverse Transcriptase Polymerase Chain Reaction; RT-qPCR - Reverse Transcriptase Quantitative Polymerase Chain Reaction; HEK293 cell - Human Embryonic Kidney cell; Vero E6 cell – cell derived from the African green monkey kidney; FRhK-4 cell – Similar epithelial cell isolated from monkey kidney.

**References**

[1] D. Wrapp, D. De Vlieger, K.S. Corbett, G.M. Torres, N. Wang, W. Van Breedam, K. Roose, L. van Schie, V.-C.C.-R. Team, M. Hoffmann, S. Pohlmann, B.S. Graham, N. Callewaert, B. Schepens, X. Saelens, and J.S. McLellan, Structural Basis for Potent Neutralization of Betacoronaviruses by Single-Domain Camelid Antibodies. Cell 181 (2020) 1004-1015 e15.

[2] L. Hanke, L. Vidakovics Perez, D.J. Sheward, H. Das, T. Schulte, A. Moliner-Morro, M. Corcoran, A. Achour, G.B. Karlsson Hedestam, B.M. Hallberg, B. Murrell, and G.M. McInerney, An alpaca nanobody neutralizes SARS-CoV-2 by blocking receptor interaction. Nat Commun 11 (2020) 4420.

[3] X. Wu, L. Cheng, M. Fu, B. Huang, L. Zhu, S. Xu, H. Shi, D. Zhang, H. Yuan, W. Nawaz, P. Yang, Q. Hu, Y. Liu, and Z. Wu, A potent bispecific nanobody protects hACE2 mice against SARS-CoV-2 infection via intranasal administration. Cell Rep 37 (2021) 109869.

[4] P. Pymm, A. Adair, L.J. Chan, J.P. Cooney, F.L. Mordant, C.C. Allison, E. Lopez, E.R. Haycroft, M.T. O'Neill, L.L. Tan, M.H. Dietrich, D. Drew, M. Doerflinger, M.A. Dengler, N.E. Scott, A.K. Wheatley, N.A. Gherardin, H. Venugopal, D. Cromer, M.P. Davenport, R. Pickering, D.I. Godfrey, D.F.J. Purcell, S.J. Kent, A.W. Chung, K. Subbarao, M. Pellegrini, A. Glukhova, and W.H. Tham, Nanobody cocktails potently neutralize SARS-CoV-2 D614G N501Y variant and protect mice. Proc Natl Acad Sci U S A 118 (2021).

[5] T. Guttler, M. Aksu, A. Dickmanns, K.M. Stegmann, K. Gregor, R. Rees, W. Taxer, O. Rymarenko, J. Schunemann, C. Dienemann, P. Gunkel, B. Mussil, J. Krull, U. Teichmann, U. Gross, V.C. Cordes, M. Dobbelstein, and D. Gorlich, Neutralization of SARS-CoV-2 by highly potent, hyperthermostable, and mutation-tolerant nanobodies. EMBO J 40 (2021) e107985.

[6] G.P. Anderson, J.L. Liu, T.J. Esparza, B.T. Voelker, E.R. Hofmann, and E.R. Goldman, Single-Domain Antibodies for the Detection of SARS-CoV-2 Nucleocapsid Protein. Anal Chem 93 (2021) 7283-7291.

[7] J.F. Li, L. He, Y.Q. Deng, S.H. Qi, Y.H. Chen, X.L. Zhang, S.X. Hu, R.W. Fan, G.Y. Zhao, and C.F. Qin, Generation and Characterization of a Nanobody Against SARS-CoV. Virol Sin 36 (2021) 1484-1491.

[8] T.J. Esparza, N.P. Martin, G.P. Anderson, E.R. Goldman, and D.L. Brody, High affinity nanobodies block SARS-CoV-2 spike receptor binding domain interaction with human angiotensin converting enzyme. Sci Rep 10 (2020) 22370.

[9] H. Ma, W. Zeng, X. Meng, X. Huang, Y. Yang, D. Zhao, P. Zhou, X. Wang, C. Zhao, Y. Sun, P. Wang, H. Ou, X. Hu, Y. Xiang, and T. Jin, Potent Neutralization of SARS-CoV-2 by Hetero-bivalent Alpaca Nanobodies Targeting the Spike Receptor-Binding Domain. J Virol 95 (2021).

[10] K. Gu, Z. Song, P. Ma, Z. Liao, M. Yang, C. Zhou, C. Li, Y. Zhao, H. Li, X. Yang, C. Lei, and H. Wang, A Novel Nanobody-Horseradish Peroxidase Fusion Based-Competitive ELISA to Rapidly Detect Avian Corona-Virus-Infectious Bronchitis Virus Antibody in Chicken Serum. Int J Mol Sci 23 (2022).

[11] T. Li, B. Zhou, Y. Li, S. Huang, Z. Luo, Y. Zhou, Y. Lai, A. Gautam, S. Bourgeau, S. Wang, J. Bao, J. Tan, D. Lavillette, and D. Li, Isolation, characterization, and structure-based engineering of a neutralizing nanobody against SARS-CoV-2. Int J Biol Macromol 209 (2022) 1379-1388.

[12] L. Hanke, D.J. Sheward, A. Pankow, L.P. Vidakovics, V. Karl, C. Kim, E. Urgard, N.L. Smith, J. Astorga-Wells, S. Ekstrom, J.M. Coquet, G.M. McInerney, and B. Murrell, Multivariate mining of an alpaca immune repertoire identifies potent cross-neutralizing SARS-CoV-2 nanobodies. Sci Adv 8 (2022) eabm0220.

[13] T. Li, B. Zhou, Z. Luo, Y. Lai, S. Huang, Y. Zhou, Y. Li, A. Gautam, S. Bourgeau, S. Wang, J. Bao, J. Tan, D. Lavillette, and D. Li, Structural Characterization of a Neutralizing Nanobody With Broad Activity Against SARS-CoV-2 Variants. Front Microbiol 13 (2022) 875840.

[14] J. Xu, K. Xu, S. Jung, A. Conte, J. Lieberman, F. Muecksch, J.C.C. Lorenzi, S. Park, F. Schmidt, Z. Wang, Y. Huang, Y. Luo, M.S. Nair, P. Wang, J.E. Schulz, L. Tessarollo, T. Bylund, G.Y. Chuang, A.S. Olia, T. Stephens, I.T. Teng, Y. Tsybovsky, T. Zhou, V. Munster, D.D. Ho, T. Hatziioannou, P.D. Bieniasz, M.C. Nussenzweig, P.D. Kwong, and R. Casellas, Nanobodies from camelid mice and llamas neutralize SARS-CoV-2 variants. Nature 595 (2021) 278-282.

[15] Q. Su, W. Shi, X. Huang, Y. Wan, G. Li, B. Xing, Z.P. Xu, H. Liu, B.D. Hammock, X. Yang, S. Yin, and X. Lu, Screening, Expression, and Identification of Nanobody against SARS-CoV-2 Spike Protein. Cells 11 (2022).

[16] J.B. Weinstein, T.A. Bates, H.C. Leier, S.K. McBride, E. Barklis, and F.G. Tafesse, A potent alpaca-derived nanobody that neutralizes SARS-CoV-2 variants. iScience 25 (2022) 103960.

[17] G. Esposito, Y. Hunashal, M. Percipalle, T. Venit, M.M. Dieng, F. Fogolari, G. Hassanzadeh, F. Piano, K.C. Gunsalus, Y. Idaghdour, and P. Percipalle, NMR-Based Analysis of Nanobodies to SARS-CoV-2 Nsp9 Reveals a Possible Antiviral Strategy Against COVID-19. Adv Biol (Weinh) 5 (2021) e2101113.

[18] M. Gransagne, G. Ayme, S. Brier, G. Chauveau-Le Friec, V. Meriaux, M. Nowakowski, F. Dejardin, S. Levallois, G. Dias de Melo, F. Donati, M. Prot, S. Brule, B. Raynal, J. Bellalou, P. Goncalves, X. Montagutelli, J.P. Di Santo, F. Lazarini, P. England, S. Petres, N. Escriou, and P. Lafaye, Development of a highly specific and sensitive VHH-based sandwich immunoassay for the detection of the SARS-CoV-2 nucleoprotein. J Biol Chem 298 (2022) 101290.

[19] R. Maeda, J. Fujita, Y. Konishi, Y. Kazuma, H. Yamazaki, I. Anzai, T. Watanabe, K. Yamaguchi, K. Kasai, K. Nagata, Y. Yamaoka, K. Miyakawa, A. Ryo, K. Shirakawa, K. Sato, F. Makino, Y. Matsuura, T. Inoue, A. Imura, K. Namba, and A. Takaori-Kondo, A panel of nanobodies recognizing conserved hidden clefts of all SARS-CoV-2 spike variants including Omicron. Commun Biol 5 (2022) 669.

[20] J. Huo, H. Mikolajek, A. Le Bas, J.J. Clark, P. Sharma, A. Kipar, J. Dormon, C. Norman, M. Weckener, D.K. Clare, P.J. Harrison, J.A. Tree, K.R. Buttigieg, F.J. Salguero, R. Watson, D. Knott, O. Carnell, D. Ngabo, M.J. Elmore, S. Fotheringham, A. Harding, L. Moynie, P.N. Ward, M. Dumoux, T. Prince, Y. Hall, J.A. Hiscox, A. Owen, W. James, M.W. Carroll, J.P. Stewart, J.H. Naismith, and R.J. Owens, A potent SARS-CoV-2 neutralising nanobody shows therapeutic efficacy in the Syrian golden hamster model of COVID-19. Nat Commun 12 (2021) 5469.

[21] Z. Ma, T. Wang, Z. Li, X. Guo, Y. Tian, Y. Li, and S. Xiao, A novel biotinylated nanobody-based blocking ELISA for the rapid and sensitive clinical detection of porcine epidemic diarrhea virus. J Nanobiotechnology 17 (2019) 96.

[22] L.C. Shriver-Lake, J.L. Liu, D. Zabetakis, V.A. Sugiharto, C.R. Lee, G.N. Defang, S.L. Wu, G.P. Anderson, and E.R. Goldman, Selection and Characterization of Anti-Dengue NS1 Single Domain Antibodies. Sci Rep 8 (2018) 18086.

[23] J. Hruskovicova, K. Bhide, P. Petrouskova, Z. Tkacova, E. Mochnacova, J. Curlik, M. Bhide, and A. Kulkarni, Engineering the Single Domain Antibodies Targeting Receptor Binding Motifs Within the Domain III of West Nile Virus Envelope Glycoprotein. Front Microbiol 13 (2022) 801466.

[24] T. Delfin-Riela, M. Rossotti, R. Alvez-Rosado, C. Leizagoyen, and G. Gonzalez-Sapienza, Highly Sensitive Detection of Zika Virus Nonstructural Protein 1 in Serum Samples by a Two-Site Nanobody ELISA. Biomolecules 10 (2020).

[25] H. Duan, Z. Ma, L. Xu, A. Zhang, Z. Li, and S. Xiao, A novel intracellularly expressed NS5B-specific nanobody suppresses bovine viral diarrhea virus replication. Vet Microbiol 240 (2020) 108449.

[26] J.L. Liu, D. Zabetakis, C.L. Gardner, C.W. Burke, P.J. Glass, E.M. Webb, L.C. Shriver-Lake, G.P. Anderson, J. Weger-Lucarelli, and E.R. Goldman, Bivalent single domain antibody constructs for effective neutralization of Venezuelan equine encephalitis. Sci Rep 12 (2022) 700.

[27] J.L. Liu, L.C. Shriver-Lake, D. Zabetakis, G.P. Anderson, and E.R. Goldman, Selection and characterization of protective anti-chikungunya virus single domain antibodies. Mol Immunol 105 (2019) 190-197.

[28] J.L. Liu, L.C. Shriver-Lake, D. Zabetakis, E.R. Goldman, and G.P. Anderson, Selection of Single-Domain Antibodies towards Western Equine Encephalitis Virus. Antibodies (Basel) 7 (2018).

[29] I.B. Esmagambetov, D.V. Shcheblyakov, D.A. Egorova, O.L. Voronina, A.A. Derkaev, D.V. Voronina, O. Popova, E.I. Ryabova, D.N. Shcherbinin, E.I. Aksenova, A.N. Semenov, M.S. Kunda, N.N. Ryzhova, O.V. Zubkova, A.I. Tukhvatulin, D.Y. Logunov, B.S. Naroditsky, S.V. Borisevich, and A.L. Gintsburg, Nanobodies Are Potential Therapeutic Agents for the Ebola Virus Infection. Acta Naturae 13 (2021) 53-63.

[30] L.J. Sherwood, and A. Hayhurst, Generating Uniformly Cross-Reactive Ebolavirus spp. Anti-nucleoprotein Nanobodies to Facilitate Forward Capable Detection Strategies. ACS Infect Dis 8 (2022) 343-359.

[31] A. Van Hout, A. Klarenbeek, V. Bobkov, J. Doijen, M. Arimont, C. Zhao, R. Heukers, R. Rimkunas, C. de Graaf, T. Verrips, B. van der Woning, H. de Haard, J.B. Rucker, K. Vermeire, T. Handel, T. Van Loy, M.J. Smit, and D. Schols, CXCR4-targeting nanobodies differentially inhibit CXCR4 function and HIV entry. Biochem Pharmacol 158 (2018) 402-412.

[32] S. Li, W. Zhang, K. Jiang, H. Shan, M. Shi, B. Chen, and Z. Hua, Nanobody against the E7 oncoprotein of human papillomavirus 16. Mol Immunol 109 (2019) 12-19.

[33] M. Togtema, G. Hussack, G. Dayer, M.R. Teghtmeyer, S. Raphael, J. Tanha, and I. Zehbe, Single-Domain Antibodies Represent Novel Alternatives to Monoclonal Antibodies as Targeting Agents against the Human Papillomavirus 16 E6 Protein. Int J Mol Sci 20 (2019).

[34] G.P. Anderson, J.L. Liu, L.C. Shriver-Lake, and E.R. Goldman, Selection and Characterization of Single-Domain Antibodies for Detection of Lassa Nucleoprotein. Antibodies (Basel) 9 (2020).

[35] E. Kinimi, S. Muyldermans, C. Vincke, S. Odongo, R. Kock, S. Parida, M. Mahapatra, and G. Misinzo, Development of Nanobodies Targeting Peste des Petits Ruminants Virus: The Prospect in Disease Diagnosis and Therapy. Animals (Basel) 11 (2021).

[36] P.J. Wichgers Schreur, S. van de Water, M. Harmsen, E. Bermudez-Mendez, D. Drabek, F. Grosveld, K. Wernike, M. Beer, A. Aebischer, O. Daramola, S. Rodriguez Conde, K. Brennan, D. Kozub, M. Sondergaard Kristiansen, K.K. Mistry, Z. Deng, J. Hellert, P. Guardado-Calvo, F.A. Rey, L. van Keulen, and J. Kortekaas, Multimeric single-domain antibody complexes protect against bunyavirus infections. Elife 9 (2020).

[37] I. Rossey, C.L. Hsieh, K. Sedeyn, M. Ballegeer, B. Schepens, J.S. McLellan, and X. Saelens, A vulnerable, membrane-proximal site in human respiratory syncytial virus F revealed by a prefusion-specific single-domain antibody. J Virol 95 (2021).

[38] W. Xue, Q. Zhao, P. Li, R. Zhang, J. Lan, J. Wang, X. Yang, Z. Xie, and S. Jiang, Identification and characterization of a novel nanobody against duck hepatitis A virus type 1. Virology 528 (2019) 101-109.

[39] Y. Cheng, Y. Hao, F. Bao, H. Zhang, Y. Liu, K. Ao, S. Fu, Q. Wu, and Z. Wang, Preparation and identification of a single domain antibody specific for adenovirus vectors and its application to the immunoaffinity purification of adenoviruses. AMB Express 12 (2022) 80.

[40] W. Ramage, T. Gaiotto, C. Ball, P. Risley, G.W. Carnell, N. Temperton, C.Y. Cheung, O.G. Engelhardt, and S.E. Hufton, Cross-Reactive and Lineage-Specific Single Domain Antibodies against Influenza B Hemagglutinin. Antibodies (Basel) 8 (2019).

[41] S. Zhu, B. Miao, Y.-Z. Zhang, W.-W. Zeng, D.-S. Wang, and S.-Q. Su, In vitro neutralization of nervous necrosis virus by a nanobody binding to the protrusion domain of capsid protein. Aquaculture 548 (2022) 737654.

[42] G. Llauger, D. Monti, M. Aduriz, E. Romao, A.D. Dumon, M.F. Mattio, A. Wigdorovitz, S. Muyldermans, C. Vincke, V. Parreno, and M. Del Vas, Development of Nanobodies against Mal de Rio Cuarto virus major viroplasm protein P9-1 for diagnostic sandwich ELISA and immunodetection. Sci Rep 11 (2021) 20013.

[43] Y. Chen, X. Wang, M. Zhang, J. Li, X. Gao, Y. Nan, Q. Zhao, E.M. Zhou, and B. Liu, Identification of two novel neutralizing nanobodies against swine hepatitis E virus. Front Microbiol 13 (2022) 1048180.
